# Direct conversion of injury-site myeloid cells to fibroblast-like cells of granulation tissue

**DOI:** 10.1038/s41467-018-03208-w

**Published:** 2018-03-05

**Authors:** Mithun Sinha, Chandan K. Sen, Kanhaiya Singh, Amitava Das, Subhadip Ghatak, Brian Rhea, Britani Blackstone, Heather M. Powell, Savita Khanna, Sashwati Roy

**Affiliations:** 1 Department of Surgery, Center for Regenerative Medicine and Cell Based Therapies, Comprehensive Wound Center, Davis Heart & Lung Institute, The Ohio State University Wexner Medical Center, Columbus, OH 43210 USA; 2 Department of Biomedical Engineering, The Ohio State University, Columbus, OH 43210 USA

## Abstract

Inflammation, following injury, induces cellular plasticity as an inherent component of physiological tissue repair. The dominant fate of wound macrophages is unclear and debated. Here we show that two-thirds of all granulation tissue fibroblasts, otherwise known to be of mesenchymal origin, are derived from myeloid cells which are likely to be wound macrophages. Conversion of myeloid to fibroblast-like cells is impaired in diabetic wounds. In cross-talk between keratinocytes and myeloid cells, miR-21 packaged in extracellular vesicles (EV) is required for cell conversion. EV from wound fluid of healing chronic wound patients is rich in miR-21 and causes cell conversion more effectively compared to that by fluid from non-healing patients. Impaired conversion in diabetic wound tissue is rescued by targeted nanoparticle-based delivery of miR-21 to macrophages. This work introduces a paradigm wherein myeloid cells are recognized as a major source of fibroblast-like cells in the granulation tissue.

## Abstract (web summary)

At the site of injury, macrophages exit their characteristic phenotype undergoing direct conversion to fibroblasts. Keratinocyte-derived miR-21, packaged in extracellular vesicles, enables such plasticity which accounts for the vast majority of all fibroblasts in the granulation tissue.

## Introduction

Transflammation introduced an intriguing link between innate immunity and cell plasticity^1,2^. At a time when much effort has been guided to glean methodological advances on how cell fate can be therapeutically directed, it has also been learnt that injury itself is a physiological trigger for cell plasticity^3,4^. During lineage reprogramming or transdifferentiation, one mature somatic cell transforms into another mature somatic cell without undergoing an intermediate pluripotent state or progenitor cell type^5^. The wound-site milieu acts as a fertile ground hosting a wide range of transdifferentiation processes^3^. Hypoxia, an inherent characteristic of the wound site, is widely recognized for its ability to facilitate cell plasticity^6^. Blood-borne myeloid cells are specifically endowed to detect sites of injury, extravasate, infiltrate, and acquire functions that are necessary for the physiological repair process^7,8^. At the wound site, these cells acquire plasticity and are known to transdifferentiate into endothelial cells supporting wound angiogenesis^9^. Current understanding of the fate of cells at the wound site and factors that guide them remains incomplete.

Monocytes and macrophages, of myeloid origin, are primarily responsible for mounting an inflammatory response at the injury site^10^. Both robust mounting of inflammation as well as timely resolution are key to successful tissue repair. The dominant fate of macrophages, following resolution of inflammation, is unclear and debated^11–14^. Egress from the injury site via lymphatic vessels^13^ and cell death^14^ are two proposed fates for injury-associated macrophages. An additional consideration here is the substantial plasticity properties of macrophages introducing the notion of a phenotypic switch at the site of injury^15^. Injury-site macrophages are not limited to switching their functional phenotype from pro-inflammatory M1 to pro-resolution M2 state^16^. Conversion of macrophages to endothelial cells, endothelial progenitor cells, or endothelial-like cells both in vitro and in vivo are evident^9,17^. In further support of robust plasticity, cells of myeloid origin can give rise to white adipocytes^18^. This study rests on our observation that at the site of injury, infiltrating myeloid cells convert to fibroblast-like cells populating the granulation tissue. The objectives of this work were to delineate the mechanism of such cell conversion at the wound site as well as to understand the significance of such conversion in wound healing. This work shows that the vast majority of the  fibroblasts at the wound site originate from cells of myeloid lineage such as macrophages. Keratinocyte-derived miR-21, packaged in extracellular vesicles, positively regulates such plasticity of wound macrophages.

## Results

### Myeloid origin of fibroblast-like cells in wound bed

**Fig. 1:**
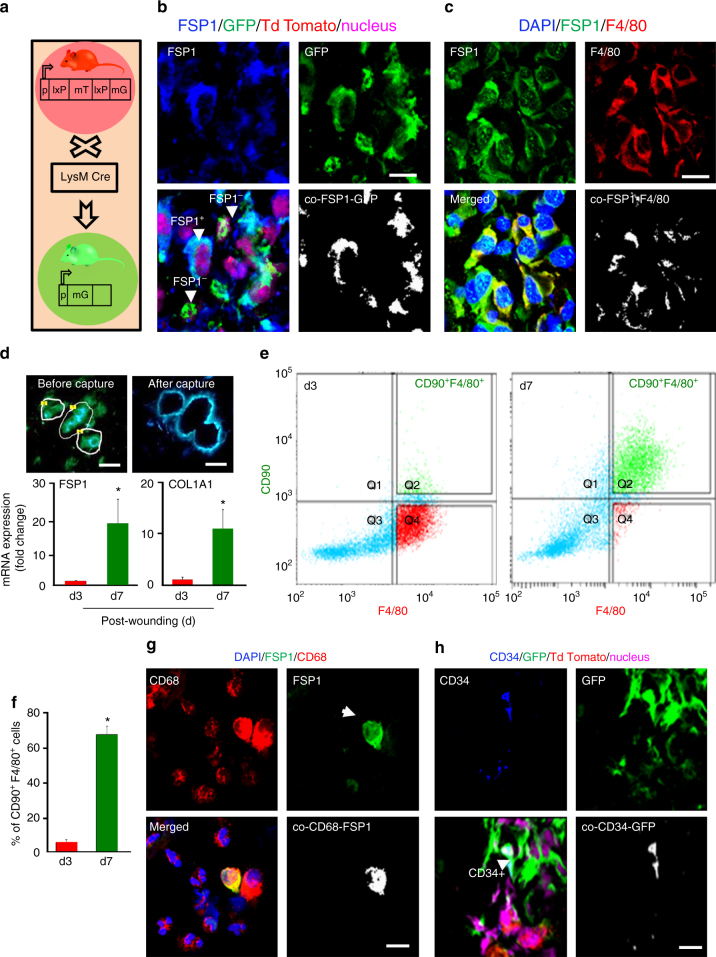
Majority of FSP1^+^ fibroblast-like cells in wound granulation tissue are of myeloid origin. **a** LysM^Cre^Rosa^mT/mG^ mice express cell membrane-localized td Tomato (red) fluorescence while cells of myeloid origin express GFP (green) fluorescence. **b** FSP1 (blue) immunostaining of LysM^Cre^Rosa^mT/mG^ mice d5 wound. Colocalization was performed using Olympus Fluoview® software. Estimated (65 ± 5%) FSP1^+^ cells (blue) were GFP^+^ demonstrating their myeloid origin, *n* = 5, scale bar = 10 µm. **c** Macrophage (F4/80, red) and fibroblast (FSP1, green) immunostaining at wound-edge tissue (d5) from C57BL/6 mice, *n* = 5, scale bar = 10 µm. **d** Laser capture micro-dissected (LCM) GFP^+^ cells from full thickness excisional wounds on d3 (early inflammation phase) or d7 (inflammation resolution phase) post-wounding (PW), scale bar = 10 µm. qRT-PCR of LCM-captured GFP^+^ cells showed increased expression of FSP1 and Col1A1 at d7, *n* = 5, Student’s *t* test *p* < 0.05. **e**, **f** Multicolor flow cytometry of CD11b^+^ of wound macrophages on d3 and d7 PW with the F4/80 (macrophage, *x*-axis) and CD90 (fibroblast marker, *y*-axis) displayed increase (68 ± 2%) in F4/80^+^CD90^+^ cells on d7 PW, *n* = 4, Student’s *t* test *p* < 0.05. **g** Chronic wound fluid derived from human subjects contain CD68^+^ FSP1^+^ cells, scale bar = 10 µm. **h** Fibrocyte marker CD34 (blue), immune-staining of LysM^Cre^Rosa^mT/mG^ mice d5 wound tissue. Estimated (15 ± 2%) of CD34^+^ cells (blue) were GFP^+^, *n* = 5, scale bar = 10 µm. Data presented as ±SD

To determine the fate of extravasated macrophages at the wound site, we developed tdTomatoLysMGFP (LysMcre/Gt (ROSA)6Sortm4(ACTB-tdTomato,-EGFP)Luo/J), referred to as LysM^Cre^-Rosa^mT/mG^mice (Fig. 1a**)**. These mice express cell membrane localized red (tdTomato) fluorescence in all cells/tissue. Cells of myeloid lineage express membrane-localized GFP^19^. Cre-recombinase regulated by LysM promoter directs the expression of Cre in activated myelomonocytic cells^20^. Vast majority (65 ± 5%) population of Fibroblast-Specific Protein 1 (FSP1)^+^ cells (blue) at the wound-edge tissue were detected to be of myeloid lineage. FSP1 had been reported to be marker of fibroblast^21^. These myeloid cells presented fibroblast-like phenotype (Fig. 1b). The possibility that these fibroblast-like cells were granulocytes was categorically ruled out based on lack of immunostaining with Myeloperoxidase (MPO) antibody. MPO^+^/FSP1^+^ cells were not detected at the wound edge (Supplementary Fig. 1a). In support of the notion that wound-site fibroblast-like cells originated from wound-site differentiated macrophages, it was observed that 68% of all FSP1^+^ cells at the wound granulation tissue were F4/80^+^ (Fig. 1c). Considering the related lineage tracing observation that 65% of all FSP1^+^ cells were of myeloid origin, it is reasonable to conclude that transitioning wound macrophages represent a major source of wound-site fibroblast-like cells. Further support to this notion was provided by immunostaining for pan fibroblast marker platelet-derived growth factor receptor α (PDGFRα)^22^ (Supplementary Fig. 1b). Taken together, these observations point toward a wound macrophage to fibroblast-like cell transition.

Laser capture microdissection studies of LysM^Cre^-Rosa^mT/mG^ mice enabled the collection of GFP^+^ cells of myeloid lineage from the wound edge followed by the study of fibroblast-specific genes. The expression of FSP1 as well as that of fibroblast-abundant Collagen 1 A1 (COL1A1) gene was significantly higher in d7 compared to that in d3. This revealed that most of the said transition of macrophages to fibroblast-like cells take place during the acute inflammatory phase (Fig. 1d). Flow cytometry of CD11b^+^ wound monocytes showed that in an early d3 time point, although none of the monocytes co-stained for fibroblast marker CD90^23,24^, a small population of F4/80^+^ macrophages demonstrated an early transition by testing positive for CD90 (Fig. 1e). On d7, a vast majority (68 ± 2.3%, *n* = 4) of all mature wound macrophage (F4/80^+^) presented as fibroblast-like by testing positive for the co-presence of CD90 (Fig. 1e, f). Consistent with these observations in experimental animal models, CD68^+^ FSP1^+^ fibroblast-like cells were detected in the wound fluid isolated from chronic wound patients (Fig. 1g).

Circulating fibrocytes (CD34^+^/Col1A^+^) are also of myeloid origin and could contribute to the fibroblast pool at the wound-site^25^. In wounds of LysM^Cre^-Rosa^mT/mG^ mice, only 15% of all GFP^+^ cells were CD34^+^ (Fig. 1h). Under the same conditions, 65–68% were fibroblast-like cells as manifested by GFP^+^/FSP1^+^ (Fig. 1a–f). Thus, it is estimated that among all fibroblast-like cells under such acute inflammatory conditions, a vast majority of the cells are represented by non-fibrocyte myeloid cells. In support of the contention that these cells originated from mature wound macrophage, transition to fibroblast-like cell was impaired in LysM^Cre^-Rosa^mT/mG^ mice treated with clodronate liposomes (Supplementary Fig. 1c). To address a related confounding factor, the possibility of cell fusion contributing toward GFP^+^/FSP1^+^ in wounds of LysM^Cre^-Rosa^mT/mG^mice was ruled out by cell ploidy analyses (Supplementary Fig. 1d). In addition to the expression of LysM promoter in cells of myeloid lineage, LysM is also reported in subsets of neuronal and neuronal progenitor cells including subsets of peripheral neurons^26^. The current study utilizes a cutaneous wound model where, albeit in very low abundance, it is plausible that some LysM-GFP may express in the peripheral neural fiber of skin wound. In the interest of additional rigor, we performed bone marrow transplantation (BMT) experiment adopting methodology previously reported by us^19^. In this experiment, GFP^+^ BM cells from B6 ACTb-EGFP mice (Jackson #006567) were transplanted to non-EGFP C57 BL/6 mice. The presence of GFP^+^ cells colocalizing with COL1/FSP1 was evident in the granulation tissue. To test whether GFP^+^ cells with fibroblast markers are indeed macrophages, an additional macrophage marker F4/80 was used. GFP^+^ FSP^+^/COL1^+^ cells tested F4/80^+^ supporting the proposed macrophage transition (Supplementary Fig. 2a-b). BM cells were transplanted from CD90.2 B6 ACTb-EGFP mice (donor, Jackson #006567) to CD90.1 (recipient, Jackson #000406) mice. Wound macrophages were isolated on d7 post-wounding and four-color flow cytometry was performed to detect F4/80, CD90.1, CD90.2, and GFP. Presence of donor macrophages (GFP^+^ F4/80^+^) expressing fibroblast marker CD90.2 (Supplementary Fig. 2d-e) is evident. The absence of lymphoid cells in isolated wound macrophage was ruled out by co-staining the cells with CD90 and CD19 (B cells)/CD3 (T Cells)/CD127 (Innate lymphoid cells) (Supplementary Fig. 2c).

**Fig. 2:**
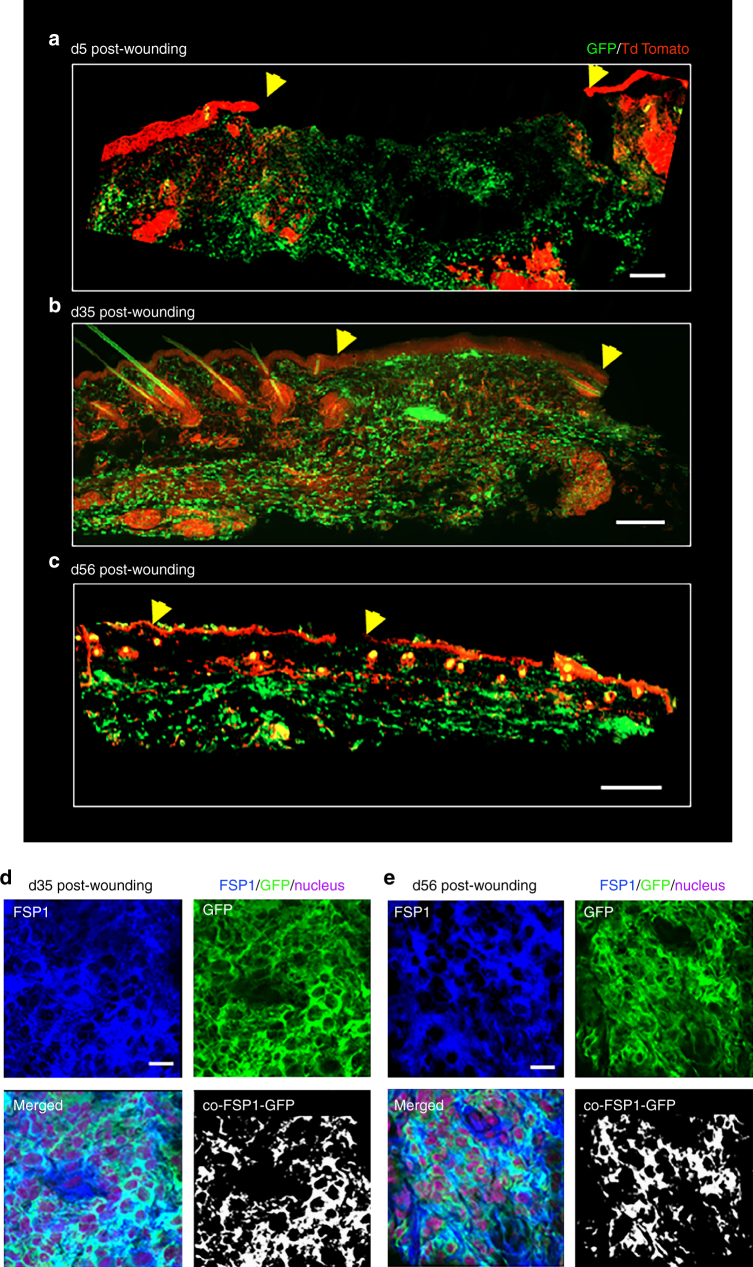
Persistence of GFP^+^ fibroblast-like cells at late remodeling phase post-wounding. **a** Conversion of wound-site macrophages (GFP^+^ green) to fibroblast-like cells was abundant, particularly closer to newly forming epidermis, at the early phase (d5). Wound edge shown in yellow arrow head, scale bar = 200 µm. **b**, **c** Such cells of myeloid origin (GFP^+^ green) were observed to be present throughout granulation tissue in later phases d35 and d56. Wound edge shown in yellow arrow head, scale bar = 200 µm. **d**, **e** FSP1 (blue) immunostaining of d35 and d56 wounds from LysM^Cre^Rosa^mT/mG^ mice. Colocalization of FSP1 with GFP^+^ cells, *n* = 5, scale bar = 10 µm

Conversion of wound-site macrophages to fibroblast-like cells was abundant, particularly closer to newly forming epidermis, at the early phase (d5) (Fig. 2a). Such cells were observed to be dispersed throughout granulation tissue in later phases (Fig. 2b-e, Supplementary Fig. 3). These observations support the contention that the converted cells prevail post-resolution and are likely to play a role in post-closure tissue remodeling.

### Transcriptome of macrophage-derived fibroblast-like cells

**Fig. 3:**
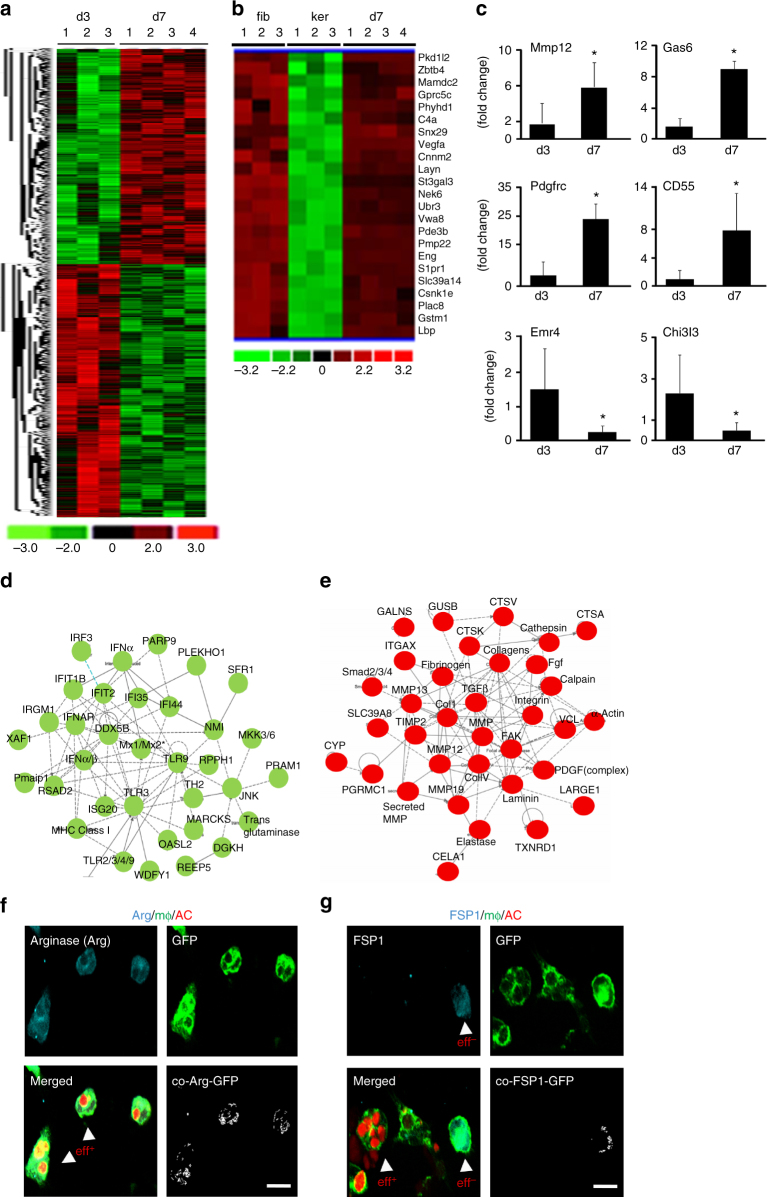
Transcriptome profiling of macrophage-derived fibroblast-like cells. **a** Heat map illustrating cluster of transcripts from CD11b^+^ F4/80^+^ wound macrophages at d3 and d7 post-wounding (PW). A total of 2757 genes were differentially expressed (*p* < 0.05, FDR). **b** Hierarchical cluster analysis of primary murine dermal fibroblast (GEO accession numbers: GSM 106139, GSM 106141, GSM 106149) and murine primary keratinocytes (GEO accession numbers: GSM 844759, GSM 844760, GSM 844761) transcriptomes obtained from Gene Expression Omnibus (GEO) database-enabled identification of a clear subset of fibroblast-specific genes differentially expressed in d7 CD11b^+^ F4/80^+^ wound macrophages. **c** qRT-PCR analysis of Mmp12, Gas6, PdgfrC, CD55, Emr4, and Chi3l3 mRNA expression identified differentially expressed in the transcriptome analysis, *n* = 5, Student’s *t* test *p* < 0.05. **d**, **e** Ingenuity**®** pathway analysis of the differentially expressed transcriptome. Pathways were scored using Fischer exact test (*p* < 0.05). **d** Downregulation of innate immune and inflammatory response genes in d7 wound macrophages compared to d3 wound macrophages. **e** Upregulation of fibroblast-specific genes in d7 wound macrophages. Pathways were scored using Fischer exact test (*p* < 0.05). **f**, **g** Efferocytosis with d3 wound macrophages (GFP^+^, green) from LysM^Cre^Rosa^mT/mG^ isolated and co-cultured with apoptotic cells (red). **f** Post-efferocytosis of apoptotic cell thymocytes (AC, red), wound macrophages (GFP, green) expressed arginase (cyan), *n* = 5, whereas **g** wound macrophages that did not participate in efferocytosis expressed FSP1 (cyan), *n* = 5, scale bar = 10 µm

Wound-derived CD11b^+^ cells sorted using flow cytometer for F4/80^+^ were subjected to GeneChip® microarray analyses at two phases of cell transition: early (day 3) and late (day 7). On day 7, transition was expected to be actively executed while on day 3 the majority wound macrophages were mostly in their native state with some early indications of commitment to transition. Out of a total of 266,220 probe sets for putative full-length mRNA tested, 2757 genes (1%) were differentially expressed in d7 compared to that in d3 (Fig. 3a). Hierarchical cluster analysis of primary murine dermal fibroblast (GEO accession numbers: GSM 106139, GSM 106141, GSM 106149) and murine primary keratinocytes (GEO accession numbers: GSM 844759, GSM 844760, GSM 844761) transcriptomes obtained from Gene Expression Omnibus (GEO) database-enabled identification of a clear subset of fibroblast-specific genes differentially expressed in d7 wound macrophages. Such evidence points toward a plausible transition of macrophages to fibroblast-like cells from days 3 to 7 (Fig. 3b). The top 10 differentially expressed genes, ranked based on fold-change data, were validated using quantitative real-time PCR (qRT-PCR) analysis (Fig. 3c, Supplementary Fig. 4b, c). Ingenuity^®^ pathway analysis of differentially expressed transcripts recognized the downregulation of gene networks that are functionally related to immune and inflammatory response (Fig. 3d). In contrast, fibroblast-specific gene networks were upregulated (Fig. 3e). Taken together, the global gene expression profiling and pathway analyses points toward a transition involving overexpression of fibroblast-specific while macrophage-related genes were downregulated.

Efferocytosis, or engulfment of apoptotic cells, is an inherent function of wound macrophages^27^. Successful efferocytosis may play a critical role in advancing pro-inflammatory M1 to reparative M2 macrophage subsets^11,19,28^. Induced expression of arginase is a hallmark of the post-efferocytotic macrophage^29^ (Fig. 3f). Of particular interest is our observation that wound macrophages that did not participate in the process of efferocytosis lent themselves to transition into FSP1^+^ fibroblast-like cells (Fig. 3g). Indeed, efferocytosis-impaired wound macrophages from milk fat globule-EGF factor 8 (MFG-E8)^−/^^−^ mice^19^ demonstrated evidence of increased transition to fibroblast-like cells (Supplementary Fig. 4a).

**Fig. 4:**
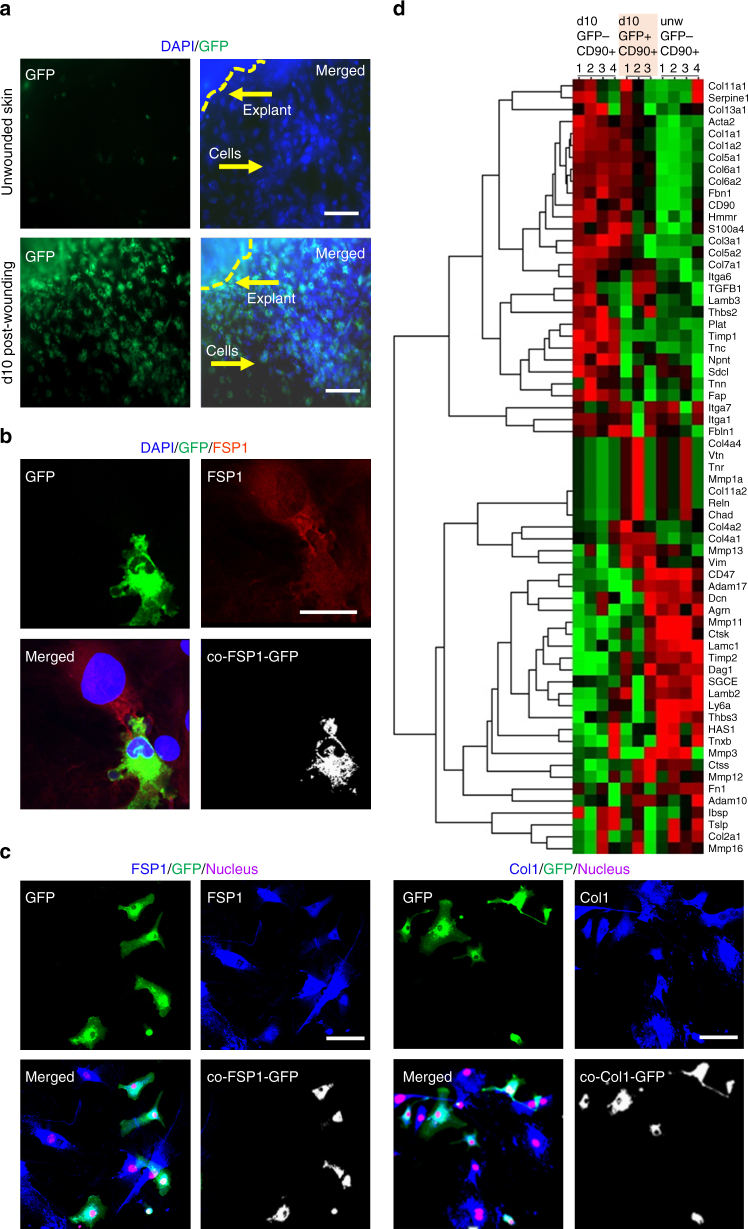
Isolation of macrophage-derived fibroblast-like cells from wound-derived explants. **a** Primary dermal fibroblasts isolated from skin or d10 post-wounding tissue of LysM^Cre^Rosa^mT/mG^ mice. Scale bar = 50 µm. **b** GFP^+^ FSP^+^ fibroblasts interlinked with GFP^−^ FSP^+^ fibroblast isolated from d10 wound explants. Scale bar = 20 µm. **c** (left) Immunostaining of GFP^+^ (myeloid, green) and FSP1 (fibroblast, blue) cells in d10 wound explants skin cultures *n* = 5, scale bar = 50 µm. Colocalization of the GFP^+^ cells (green) with FSP1^+^ (blue) demonstrating their myeloid origin. (right) Immunostaining of GFP ^+^ (myeloid, green) and Col1 (fibroblast, blue) cells in d10 wound explants skin cultures *n* = 5, scale bar = 50 µm. Multiple fibroblast-specific staining (FSP1/Col1) demonstrated the purity (>99%, fibroblasts) of the explant culture. **d** Heat map illustrating clusters of transcripts from GFP^−^CD90^+^ or GFP^+^CD90^+^ primary fibroblasts isolated from d10 wound explants. Additionally, GFP^-^CD90^+^ primary fibroblast were isolated from unwounded skin. Gene expression profiling of 70 genes relating to ECM/fibroblast was performed using NanoString nCounter system. The expression pattern of GFP^−^CD90^+^ or GFP^+^CD90^+^ primary fibroblasts from d10 wound explants were largely similar, i.e., not significantly different (*t*-test with Bonferroni correction) but was distinctly different from unwounded skin GFP^-^CD90^+^ primary fibroblasts

An additional line of evidence testing whether wound-site macrophages convert to fibroblast-like cells was obtained from a wound explant system supplemented with fibroblast-selective media allowing isolation of purified primary fibroblasts^30^. Isolation of CD90^+^/FSP1^+^ wound fibroblasts using this approach showed an abundance of GFP^+^ cells indicating myeloid lineage (Fig. 4a, Supplementary Fig. 5). Interestingly, such myeloid-lineage fibroblasts established interlinks^31^ with the FSP^+^/GFP^−^ non-myeloid fibroblasts (Fig. 4b). Cognizant of the fact that CD90 is also expressed by the other cells including cells of lymphoid origin^32^, additional experiments were aimed at determining the purity of the explant culture using multiple fibroblast-specific staining (Fig. 4c). Presence of any major lymphoid origin cells in CD90^+^ sorted explant culture cells was ruled out using multicolor flow cytometry (Supplementary Fig. 6 a,b).

Flow sorting of myeloid-lineage wound-site fibroblasts (GFP^+^/CD90^+^) of LysM^Cre^Rosa^mT/mG^ mice enabled further characterization of these cells. Comparison of gene expression profile of myeloid fibroblast-like cells to reference non-myeloid fibroblasts recognized the parallel between the two types of cell as it relates to expression of ECM/fibroblast-specific genes (Fig. 4d). This line of evidence lends compelling support to the notion, developed by other lines of evidence in this work, that at the wound-site macrophages convert to fibroblasts.

### miR-21 is required for wound macrophage conversion

**Fig. 5:**
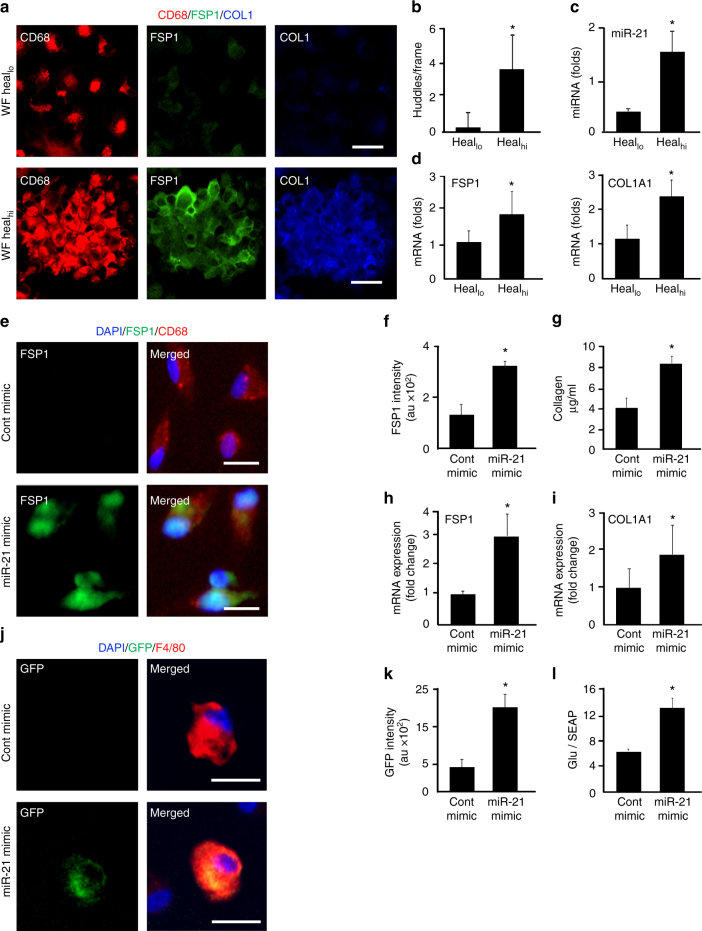
Conversion of wound macrophages to fibroblast-like cells require miR-21. **a** MDM were polarized to pro-inflammatory M1 with LPS and IFNγ. Treatment of polarized MDM (CD68^+^, red) with fluids from either healing wounds (heal_hi_) or non-healing wounds (heal_lo_) for 72 h. FSP1 (green) and COL1 (blue) were determined using immunostaining, scale bar = 20 µm. **b** The treatment with heal_hi_ wound fluid resulted in huddling. **c **qRT-PCR analysis of miR-21 in fluids from heal_hi_ and heal_lo_ wounds *n* = 5, Student’s *t* test *p* < 0.05. **d** qRT-PCR analysis of FSP1 and Col1A1 expressions in MDM treated with heal_hi_ or heal_lo_ wound fluids, *n* = 5, Student’s *t* test *p* < 0.05. **e**, **f** miR-21 or control mimics were delivered to M1-polarized MDM followed by **e** immunostaining with CD68 (macrophage, red) and FSP1 (fibroblast, green), scale bar = 10 µm. **f** Quantification of FSP1 intensity in control and miR-21 mimic-transfected MDMs, *n* = 5, Student’s *t* test *p* < 0.05. **g** miR-21 mimic-transfected MDM cells displayed increased collagen production as measured using Sircoll assay, *n* = 5, Student’s *t* test *p* < 0.05. **h**, **i** qRT-PCR analysis of **h** FSP1 and **i** Col1A1 in miR-21 mimic-delivered MDM, *n* = 6, Student’s *t* test *p* < 0.05. **j**, **k** miR-21 delivery induced Col1A1 promoter in wound macrophage from pOBCol3.6GFPtpz transgenic mice. The wound macrophages (red) were isolated on d3 post-wounding (inflammatory phase) followed by miR-21 or control mimic delivery. The activation of Col1A1 promoter (GFP, green) was imaged. **k** GFP intensity in control and miR-21 mimic-transfected cells, *n* = 5, Student’s *t* test *p* < 0.05, scale bar = 10 µm. **l** M1 polarized human MDM polarized to M1 were transfected with luciferase reporter vector pEZX-PG04 containing promoter for FSP1 (NM_002961) upstream of secreted Gaussia luciferase (Gluc) and secreted alkaline phosphatase (SEAP, endogenous control) along with miR-21 or control mimic delivery. Luciferase activity measured as GLuc/SEAP, *n* = 5, Student’s *t* test *p* < 0.05

In the wound milieu, cell fate is guided by micro environmental cues. For wounds, much of this cue is captured in the wound fluid^33^. In the context of macrophage conversion, do wound fluid from healing and non-healing chronic wound patients perform differentially? Human peripheral blood monocyte-derived macrophages (MDMs) polarized to M1 were treated with wound fluid obtained from healing or non-healing chronic wounds. Interestingly, wound fluid from healing but not from non-healing cases caused huddling of cells, characteristic of cell conversion^34^ (Fig. 5a, b). Indeed, such huddled cells showed signs of transition to fibroblast-like cells as evident by increased expression of FSP1 and Col1A1 (Fig. 5a, d). To understand the causative factor underlying such transition, exosomal content of wound fluid from healing patients were analyzed revealing higher abundance of miR-21 compared to that from fluid of non-healing cases (Fig. 5c). The significance of miR-21 as an active principle in the wound fluid, which advances wound macrophages to fibroblast-like cells, was heightened by our finding that delivery of miR-21 mimic to M1 macrophages induced cell conversion. Such conversion of macrophages manifested as increased FSP1 protein expression, FSP1/COL1A1 gene expression and increased collagen production as determined using Sircoll assay (Fig. 5e–i, Supplementary Fig. 7b, d). Sequestration of miR-21 from the wound fluid of healing patients markedly impaired cell conversion (Supplementary Fig. 7a). miR-21 delivery ex vivo to day 3 wound macrophages from pOBCol3.6GFPtpz tg mice, that express topaz-GFP upon COL1A1 promoter, led to promoter activation (GFP^+^, Fig. 5j, k). Furthermore, delivery of miR-21 to human M1 macrophages caused activation of FSP1 promoter (Fig. 5l). Taken together, it is evident that elevated miR-21 plays a critical role in the conversion of macrophages to fibroblast-like cells. The possibility that higher myeloid fibroblast abundance may have been caused by proliferation of converted cells, as opposed to induction of conversion, was ruled unlikely based on the observation that miR-21 mimic treatment did not induce Ki67 in converted cells (Supplementary Fig. 7c).

### miR-21 mechanisms in wound-macrophage conversion

**Fig. 6:**
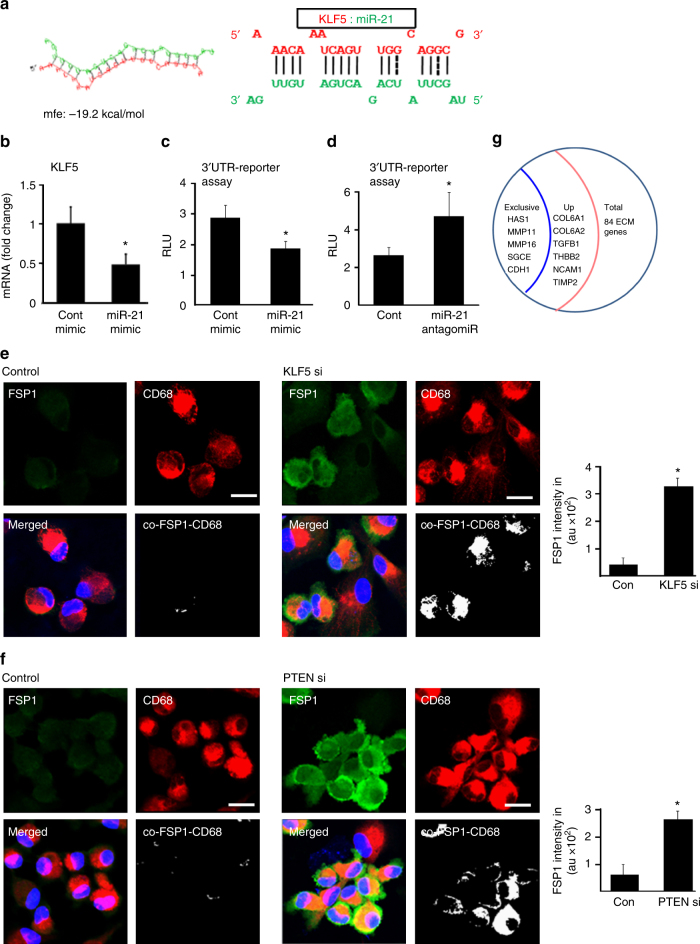
miR-21 targets KLF5 and PTEN for wound macrophage to fibroblast-like cell conversion. **a** miR-21 predicted to target the KLF5 3′UTR position 348–368 using RNAHybrid prediction algorithm. **b** qRT-PCR analysis of KLF5 expression in miR-21 transfected M1 polarized human MDM, *n* = 5, Student’s *t* test *p* < 0.05. **c**, **d** KLF5- 3′UTR luciferase reporter assay transfected with either (**c**) miR-21 mimic or (**d**) miR-21 antago-miR in M1-polarized human MDM, *n* = 6, Student’s *t* test *p* < 0.05. **e**, **f** CD68 (macrophage, red) and FSP1 (fibroblast, green) immunostained MDM transfected with (**e**) KLF5 si and (**f**) PTEN si, *n* = 6, Student’s *t* test *p* < 0.05, scale bar = 10 µm. **g** M1 polarized human MDM transfected with both PTEN si and KLF5 si were subjected to RT^2^ Profiler PCR array for human extracellular matrix and adhesion molecules. The expression of HAS1, MMP11, MMP16, SGCE, and CDH1 were present only in the double knockdown (PTEN si and KLF si) cells while it was not detected in the control-transfected group. Expressions of a total of six genes were specifically upregulated in the PTEN si/KLF5 si-treated cells as comparted to the control si-treated cells, *n* = 4, Student’s *t* test *p* < 0.05

The involvement of miR-21 in cell conversion implicates two pathways of post-transcriptional gene silencing. First, miR-21 targeted KLF-5. Bioinformatics analysis showed seed sequence for miR-21 pairing on the 3′UTR of KLF-5 (Fig. 6a). We then established KLF-5 as a direct target of miR-21 using 3′UTR reporter assays (Fig. 6b–d). Knockdown of KLF-5 using siRNA-augmented FSP1 demonstrating a critical role of KLF-5 in miR-21-mediated FSP1 and Vitronectin expression (Fig. 6e, Supplementary Fig. 8a-b). Second, miR-21 silenced PTEN. The significance of PTEN in miR-21-dependent cell conversion was established in studies demonstrating that PTEN silencing indeed augmented FSP1 and Vitronectin expression (Fig. 6f, Supplementary Fig. 8c). Furthermore, double silencing of both KLF-5 and PTEN, as would be achieved by miR-21, was potent in inducing the expression of multiple fibroblast-related ECM genes (Fig. 6g).

### Wound-site keratinocytes influence macrophage function

**Fig. 7:**
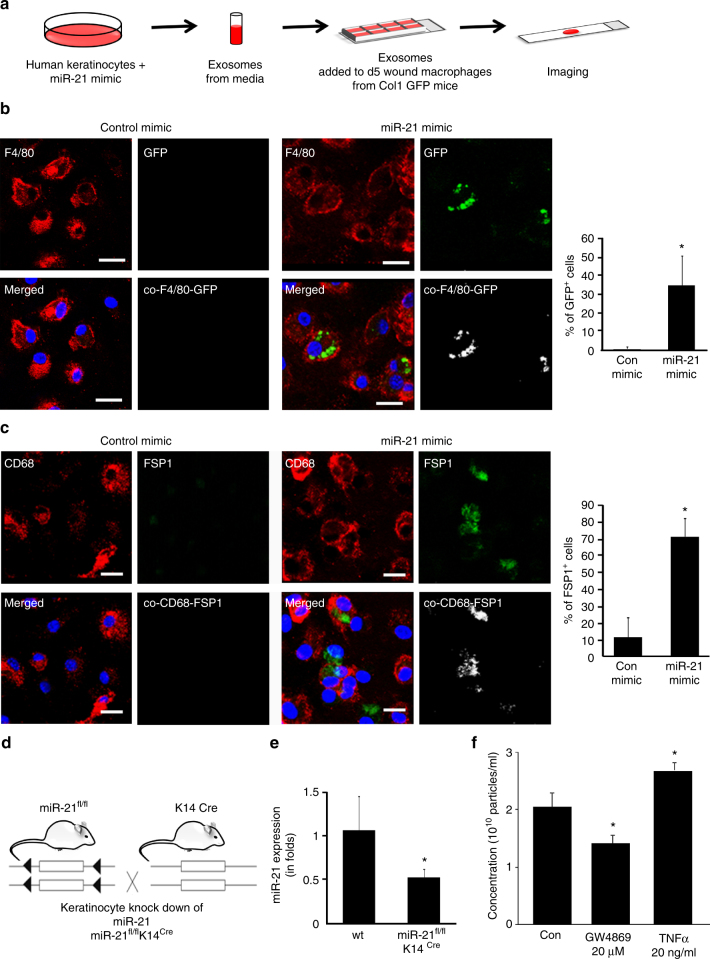
Keratinocyte to macrophage cross talk: transport of miR-21 via exosomes. **a**, **b** Day 5 wound macrophages harvested from pOBCol3.6GFPtpz transgenic mice were treated with exosomes derived from human keratinocytes (HaCaT) that were transfected with miR-21 or control mimic. Exosomes derived from miR-21 mimic-delivered keratinocytes induced Col1A1 promoter activity (GFP^+^ green cells), *n* = 5, Student’s *t* test *p* < 0.05, scale bar = 10 µm. **c** CD68 (macrophage, red) and FSP1 (fibroblast, green) immunostained MDM co-cultured with human keratinocytes (HaCaT) in a transwell chamber where keratinocytes-transfected miR-21 or control mimic were seeded on the transwell membrane (0.22 µm), *n* = 5, Student’s *t* test *p* < 0.05, scale bar = 10 µm. **d** Schematic representation of depletion of miR-21 from keratinocytes using Cre-recombinase under the control of keratin 14 promoter. **e** qRT-PCR analysis of miR-21 expression from wound fluid exosomes isolated from miR-21^fl/fl^ K14^Cre^ mice or corresponding wt (miR-21^fl/fl^) mice, *n* = 5, Student’s *t* test *p* < 0.05. **f** The exosome concentration measured from human keratinocytes (HaCaT) treated with exosome inhibitor GW4869 or inflammatory cytokine TNFα, *n* = 6, Student’s *t* test *p* < 0.05

Keratinocyte-derived exosomes are rich in miR cargo^35^. miR-21 is abundantly expressed in epithelial cells^36^. At the wound site, do keratinocyte miR-21 influence macrophage function? Exosomes isolated from human HaCaT keratinocytes enriched with miR-21 mimic directly activated the Col1A1 promoter of wound macrophage (Fig. 7a, b). Furthermore, miR-21-enriched keratinocytes induced FSP1 expression in wound macrophages placed in a chamber separated by a cell-impermeable transmembrane (Fig. 7c). To test the significance of keratinocyte miR-21 on wound macrophage function, miR-21^fl/fl^ K14^Cre^ mice were developed to conditionally deplete miR-21 from keratin14^+^(K14^+^) cells (Fig. 7d, Supplementary Fig. 9a). This depletion resulted in significant lowered level of miR-21 levels in exosomes of the wound fluid recognizing keratinocytes as a contributor of miR-21 to the wound fluid environment (Fig. 7e). Cellular EV release is regulated by micro environmental conditions such as inflammation. EV production by the skin during the inflammatory phase of wound healing was six folds higher than that of the intact skin (Supplementary Fig. 9c). Keratinocyte origin of such EV was tested by tracking keratinocyte specific proteins as component of EV cargo (Supplementary Fig. 9d). This work recognizes TNFα, a key pro-inflammatory cytokine at wound site, as a marked inducer of EV release from human keratinocytes (Fig. 7f, Supplementary Fig. 9b).

**Fig. 8:**
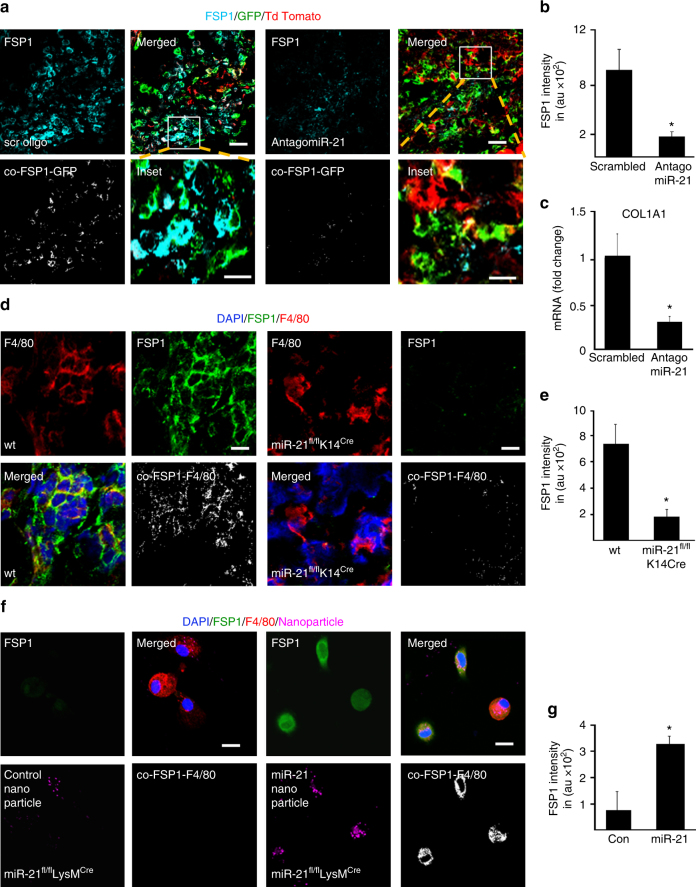
Keratinocyte-specific depletion of miR-21 impairs macrophage conversion. **a** FSP1 (cyan) immunostaining and macrophages (GFP^+^, green) colocalization in d5 wound tissue from LysM^Cre^Rosa^mT/mG^ mice where antago-miR-21 or scrambled oligo were topically delivered to the wounds, scale bar = 50 µm, scale bar of inset = 10 µm. **b** FSP1 intensity in scrambled and antago-miR-21 delivered mice, *n* = 5, Student’s *t* test *p* < 0.05. **c** q-RT-PCR analyisis of COL1A1 expression in LysM^Cre^Rosa^mT/mG^ mice d5 wound tissues scrambled and antago-miR-21, *n* = 5, Student’s *t* test *p* < 0.05. **d** Macrophage (F4/80, red) and fibroblast (FSP1, green) immunostaining of d5 wound tissue from wt and miR-21^fl/fl^K14^Cre^ mice, *n* = 5, Student’s *t* test *p* < 0.05, scale bar = 10 µm. **e** FSP1 intensity analysis in wt(miR-21^fl/fl^) and miR-21^fl/fl^ K14^Cre^ mice, *n* = 5, Student’s *t* test *p* < 0.05. **f** F4/80 (macrophage, red) and FSP1 (fibroblast, green) immunostained wound macrophages isolated from miR-21^fl/fl^LysM^Cre^mice. Rescue of FSP1 expression (green) with nanoparticles mediated miR-21 (magenta) delivery, scale bar = 10 µm. **g** FSP1 intensity in wound macrophages delivered with miR-21 compared to control mimic, *n* = 5, Student’s *t* test *p* < 0.05

**Fig. 9:**
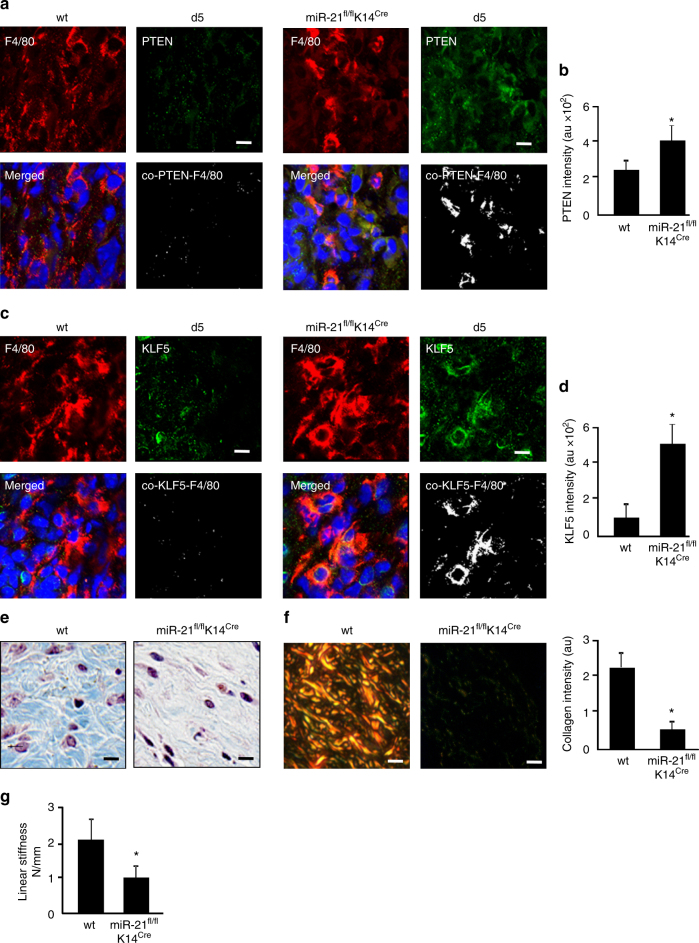
Keratinocyte-specific depletion of miR-21 impairs macrophage transition and healing. **a** Macrophage (F4/80, red) and KLF5 (green) immunostaining of d5 wound tissue from wt and miR-21^fl/fl^K14^Cre^ mice, *n* = 5, scale bar = 10 µm. **b** Quantification of KLF5 expression in wt (miR-21^fl/fl^) and miR-21^fl/fl^ K14^Cre^ mice, *n* = 5, Student’s *t* test *p* < 0.05. **c** Macrophage (F4/80, red) and PTEN (green) immunostaining of d5 wound tissue from wt and miR-21^fl/fl^ K14^Cre^ mice, *n* = 6, scale bar = 10 µm. **d** Quantification of PTEN expression in wt (miR-21^fl/fl^) and miR-21^fl/fl^ K14^Cre^ mice, *n* = 6, *p* < 0.05. **e** Masson's Trichrome staining showing collagen (blue) levels in d10 healed wounds in wt(miR-21^fl/fl^) and miR-21^fl/fl^ K14^Cre^ mice, *n* = 5, Student’s *t* test *p* < 0.05, scale bar = 10 µm. **f** Picro-sirius staining showing collagen (yellow) levels of d10 healed wounds in wt(miR-21^fl/fl^) and miR-21^fl/fl^ K14^Cre^ mice, *n* = 5, *p* < 0.05, scale bar = 10 µm. Bar graph presents quantitation of blue pixels (collagen) in Masson’s trichrome in wt (miR-21^fl/fl^) and miR-21^fl/fl^ K14^Cre^ mice, *n* = 4, Student’s *t* test *p* < 0.05. **g** Linear skin stiffness measurement of healed wounds on d14 post-wounding from miR-21 ^fl/fl^K14^Cre^and wt (miR-21^fl/fl^) mice, *n* = 6, Student’s *t* test *p* < 0.05

To test the functional significance of keratinocyte miR-21 on wound macrophage function in lineage tracing LysM^Cre^Rosa^mT/mG^ mice, wound-edge tissue miR-21 was silenced using anti-miR21 LNA (Supplementary Fig.10a,b). Silencing of miR-21 in the wound-edge skin significantly blunted macrophage to fibroblast-like cell transition at wound-site as manifested by lowered GFP^+^/FSP^+^ cells (Fig. 8a–c, Supplementary Fig. 10a). Complimentary data to test the robustness of these findings were obtained from miR-21^fl/fl^ K14^Cre^mice where miR-21 was depleted in K14^+^ cells of these mice. Depletion of keratinocyte-specific miR-21 led to marked attenuation of FSP^+^/Col1^+^/F4/80^+^ cells at the wound site underscoring the key contribution of keratinocyte-specific miR-21 in macrophage to fibroblast transition (Fig. 8d, e, Supplementary Fig. 10c). Interestingly, the targets of miR-21, KLF-5, and PTEN were markedly augmented in the K14^+^ cells of these mice (Fig. 9a–d). Blunting of macrophage to fibroblast-like cell transition at the wound site caused marked deficiency in wound granulation tissue collagen content **(**Fig. 9e–g, Supplementary Fig. 11a). As expected, wound repair limited by inadequate collagen content was manifested by compromised stiffness of the repaired skin (Fig. 9g). Taken together, wound-edge keratinocyte miR-21 enables macrophage to fibroblast-like cell transition and physiological collagen deposition at the wound site.

### The wound microenvironment guides macrophage conversion

Depletion of miR-21 in cells of myeloid origin at the wound site, as in miR-21^fl/fl^LysM^Cre^ mice, did not influence macrophage-to-fibroblast transition (Supplementary Fig. 10d). However, when wound macrophages were isolated from the in vivo wound milieu on day 3, transition toward fibroblasts ex vivo was compromised (Fig. 8f, g). This observation indicated that miR-21-depleted wound macrophages relied on a factor external to macrophages for their transition. We identified the missing factor to be miR-21 because addition of miR-21 to macrophages rescued transition to fibroblasts (Fig. 8f, g).

### Macrophage conversion is compromised in diabetes

**Fig. 10:**
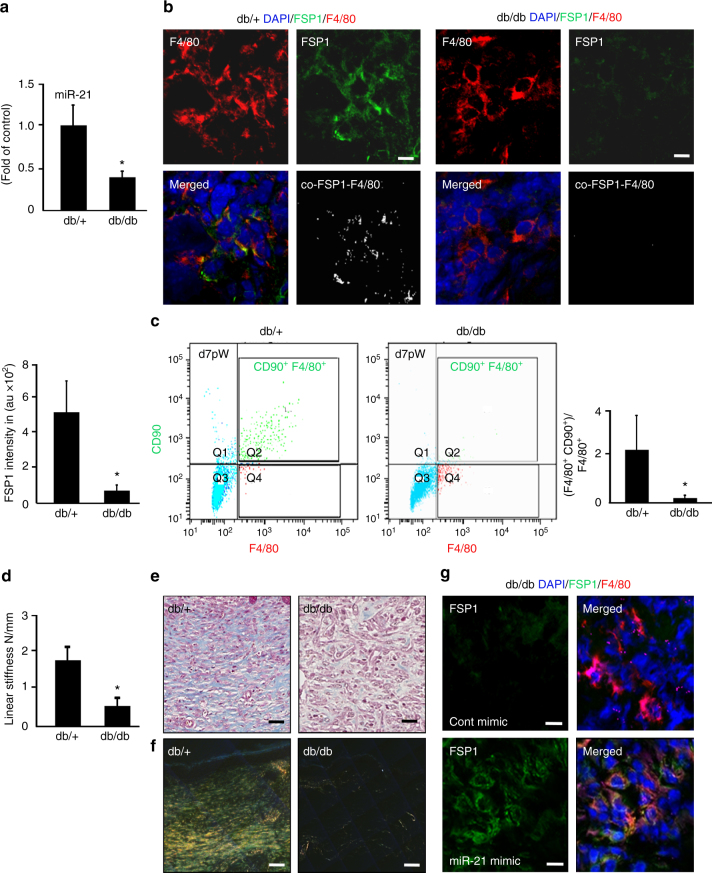
Compromised transition of wound macrophages to fibroblast-like cells in diabetic mice. **a** qRT-PCR analysis of miR-21 expression in d5 wound tissue db/db and control db/+ mice, *n* = 5, Student’s *t* test *p* < 0.05. **b** Immunostaining of F4/80 (macrophage, red) and FSP1 (fibroblast, green), immunostaining of d5 wound tissue from db/+ and db/db mice. *n* = 5, *p* < 0.05. FSP1 intensity is expressed in arbitrary units, *n* = 5, Student’s *t* test *p* < 0.05, scale bar = 10 µm. **c** Multicolor flow cytometry of CD11b^+^ of d7 wound macrophages from db/+ and db/db mice F4/80 (macrophage, *x*-axis) and CD90 (fibroblast marker, *y*-axis) of db/+ and db/db mice, *n* = 5, Student’s *t* test *p* < 0.05. **d** Linear stiffness of the repaired skin in db/+ and db/db mice on d20 PW measured using mechanical tester, *n* = 6, Student’s *t* test *p* < 0.05. **e**, **f** Collagen expression as visualized using **e** Masson’s trichrome staining (collagen, blue) and **f** picrosirius staining (collagen, yellow) of healed wounds in db/+ and db/db mice, *n* = 5, Student’s *t* test *p* < 0.05, scale bar = 20 µm. **g** FSP1 (green) and F4/80(red) immunostained d5 wounds from db/db mice delivered with miR-21 via nanoparticles. FSP1 intensity presented in arbitrary units, *n* = 5, Student’s *t* test *p* < 0.05 compared to db/db mice delivered with control mimic, scale bar = 10 µm

In Lepr db/db type 2 diabetic (T2D) mice, induction of wound-site miR-21 is non-responsive to wounding. Such impairment is evident in both early (d5) and late (d20) phases on wound healing (Fig. 10a, Supplementary Fig. 12b). Secondary to such impairment, levels of miR-21 targets KLF5 and PTEN failed to dip following wounding as noted in non-diabetic controls (Supplementary Fig. 12d, e). Under such conditions, FSP-1/Col1 and F4/80 colocalization studies revealed severe impairment of transition to fibroblast-like cells in diabetic mice at early (d5) as well as delayed (d20) phases of wound healing (Fig. 10b, Supplementary Figs. 12a, 13a-b). Higher abundance of non-myeloid FSP1/Col1 on d20, compared to d5, may be viewed as a futile compensatory response aimed at covering for impaired conversion of myeloid cells (Fig. 10b, Supplementary Figs. 12a, 13a, b**)**.

Flow cytometry analysis of CD11b^+^ wound monocytes from db/db mice showed a significant reduction of transitioning F4/80^+^/CD90^+^ wound macrophages (Fig. 10c). Impaired transition in diabetic wounds was followed by limited wound collagen and skin stiffness (Fig. 10d–f, Supplementary Fig. 12c). The delivery of miR-21 mimic to the diabetic wound-edge skin improved miR-21 levels and corrected transition of wound macrophages to fibroblast-like cells (Fig. 10g).

## Discussion

Cells of the myeloid lineage display considerable plasticity. Lineage tracing studies established that myeloid cells may convert to endothelial cells^9^ or white adipocytes^18^. Other lines of evidence claim that macrophages may give rise to osteoclasts^37^.This work provides first evidence not only to establish that cells of myeloid origin may convert to fibroblasts but also that the majority of fibroblasts at the wound site are contributed by such conversion. Thus, the reported conversion represents a dominant fate of blood-borne myeloid cells at the wound site. The source and characteristics of fibroblast-like cells at the site of injury has been a center of active debate^38,39^. While study in cardiac fibroblast suggest that resident fibroblast proliferate upon stress^40^, other studies have reported that blood-derived fibrocytes migrate to site of injury^41^. We have shown very low abundance of fibrocytes in granulation tissue. Therefore, it is evident that the fibroblast population at the wound site may not be accounted for simply by the hyperplasia of resident tissue fibroblasts or by fibrocytes. In this study, we showed through flow cytometry, the increased abundance of fibroblast-like cells from macrophages in d7 compared to d3. In support of our study, Ross and Beditt reported in 1961, that the fibroblast starts to appear in the granulation tissue on day 3 post-wounding and reached their peak on day 7^42^. Furthermore, early reports indicated that peritoneal macrophages and those of tumor origin express collagen 1^43,44^. In addition, FSP1 expression has been reported in a subset of inflammatory macrophages following liver injury^45^. Anecdotal evidence of macrophage to fibroblast conversion has been reported under pathological conditions of helminth infection^46^ and in cardiac as well as renal fibrosis^47,48^. These studies lacked mechanistic insight and characterization of the conversion. Genetic ablation of macrophages from the wound site diminishes the abundance of fibroblasts and compromise collagen deposition^49,50^. This work provides definitive evidence using lineage tracing and mechanistic studies recognizing blood-borne myeloid cells as the primary source of wound fibroblasts populating the granulation tissue. During the inflammatory phase, myeloid to fibroblast conversion is efficient in non-diabetics but impaired in diabetics. This latter deficit is responsible for lack of advancement of wound macrophages to their intended fibroblast fate thus causing longer retention of wound macrophages in their pro-inflammatory state. This notion is consistent with abundant literature reporting persistent inflammatory response in diabetics^27,51^. Long-term persistence in post-repair skin and elevated expression profile of fibroblast and ECM genes lend credence to the postulate that the converted cells not only help advance the wound healing process beyond the inflammatory phase but also play an active role in post-closure tissue remodeling. The physiological significance of converted fibroblasts is therefore wide ranging and is expected to rapidly unfold in the context of injury repair of specific organs.

Development of the significance of myeloid-converted fibroblast-like cells at the wound site rests on the appreciation that the production of appropriate extracellular matrix (ECM) represents a major cornerstone of the healing process^52^. Discussion on this topic is often skewed toward the scarring response, which may be viewed as a reflection of dysregulated ECM homeostasis. During the course of physiological healing, matrix metalloproteases regulate the ECM turnover to avert any such dysregulation. For example, MMP12 limits fibrosis^53^. In this work, upregulation of MMP12 in converted fibroblast-like cells and persistence of these cells until the late remodeling phase of wound healing support the contention that these cells are an integral component of physiological tissue repair and remodeling. Under conditions of diabetes, myeloid to fibroblast-like conversion was compromised. This finding is consistent with previous observations that the diabetic cutaneous wound suffer from compromised ECM production^54^. ECM provides essential physical scaffolding for the cellular constituents^55^ and in doing so advances the healing process through the physiological route of re-epithelialization, wound angiogenesis and tissue remodeling^52^. Indeed, wound fluid from healing chronic wound patients was able to convert M1-polarized human macrophages to fibroblast-like cells. Such effect was not recorded for wound fluid from non-healing chronic wound patients. As the primary contributor of wound-site ECM in the early-inflammatory phase, myeloid-converted fibroblast-like cells are a critical driver of the physiological healing response. Two earlier lines of investigation from our laboratory recognized a central role of miR-21 in determining the functional fate of wound macrophages^28^, and in regulating ECM homeostasis at the site of tissue injury^56^. Successful and adequate efferocytosis constitutes a critical cue that switches macrophages from pro-inflammatory to a pro-resolving phenotype^19,27^. This is enacted by induction of miR-21^28^. Such efferocytosis-dependent induction of miR-21 is compromised in diabetic wound macrophages. Low miR-21 in the diabetic wound tissue restrains macrophage plasticity dampening the transition to fibroblast-like cells. miR-21 has been directly implicated in cellular plasticity^57^. In this work, we recognize a direct contribution of inducible miR-21 in enabling the conversion of myeloid cells to fibroblast-like cells at the wound site via two distinct pathways. KLF5 was recognized as a novel miR-21 target in this work. KLF5 has been recently recognized as a myeloid transcription factor with a role in determining lineage choice^58^. Furthermore, silencing of KLF5 favors cell transition^59^. PTEN, the second pathway, was recognized by our laboratory as a miR-21 target in fibroblasts^56^. The lipid phosphatase PTEN plays a key role in determining macrophage cell fate^60^. Specifically, silencing of PTEN advances cell transition^61^. Of relevance in this context, elevated levels of PTEN have been reported in T2DM patients^62^. In this work, while myeloid-specific deletion of miR-21 had no influence on the above-mentioned conversion, myeloid to fibroblast-like conversion was markedly blunted in mice where miR-21 was ablated specifically in keratinocytes. These findings pointed toward a potential keratinocyte-myeloid cell cross-talk involving miR-21. Of note, such cross-talk with keratinocytes has been reported for innate immune cells^63^ as well as for melanocytes^35^. Efforts to characterize the nature of cross-talk between keratinocytes and myeloid cells at the wound site led to the finding that keratinocytes originating V) carry miR-21 as cargo to be delivered to myeloid cells. miR-21 delivery is critically important in converting wound-infiltrating myeloid cells to fibroblast-like cells. Consistent with this observation, miR-21 in the wound fluid of healing chronic wound patients was observed to be packaged in EV. In non-healing patients, wound fluid-derived EVs showed lower abundance of miR-21. Post-injury inflammation induces EV release from ketainocytes^64^. TNFα, a key pro-inflammatory cytokine at the wound site^65^, emerged as a marked inducer of EV release from human keratinocytes. EVs are known to participate in cell-to-cell communications^66^. Keratinocyte-secreted exosomes loaded with mRNA and miRNA play an important role during injury-repair process^35,67^ and cellular reprogramming^68^. We have recently reported that skin-derived EVs may achieve vasculogenic direct conversion of fibroblasts in vivo^69^. The present study assigns EV-dependent cross-talk between keratinocytes and myeloid cells at the wound site a pivotal role in cellular plasticity and somatic cell conversion central to physiological tissue repair. This work provides a novel conceptual framework to explain results that have existed in the literature for many years. FSP1^+^ macrophages have been reported in the context of tissue injury^45,70^. While they have been recognized as specific subsets of macrophage, systematic studies on the origin of  such subsets were lacking. The versatile plasticity of macrophages has been long recognized. In that vein, concepts that dominated include conversion of macrophage to endothelial and adipose cells^9,18^. This work provides a set of evidence establishing that during the acute phase of wound inflammation, conversion of wound-site macrophages to fibroblasts represents a significant event. A missing link that helped develop the current paradigm is the recognition of EVs as a major contributor to cell–cell communication at the wound site. Macrophages arrive at the wound site armed with the capability to respond to the call of the tissue microenvironment. In this case, it is the EV-delivered cue from the resident keratinocytes that direct the conversion of macrophage to fibroblasts. Taken together, findings of this work introduce a new paradigm where blood-borne myeloid cells infiltrating the wound site are recognized as a major source of fibroblast-like cells in the granulation tissue. Studies addressing specific myeloid cell populations led to evidence demonstrating direct conversion of wound-site macrophages to fibroblast-like cells. Such finding also provides a novel dimension to the currently understood fate of macrophages at the site of injury. Ability to convert myeloid to fibroblast-like cells was prominent property of the wound fluid from healing patients. Furthermore, the process of conversion was strikingly impaired under conditions of experimental diabetes. Thus, the reported conversion to fibroblast-like cells is likely to be an integral component of the physiological repair process. A single miR, miR-21, was observed to be critically important in enabling the above-mentioned cell conversion. Importantly, myeloid cells had to rely on miR-21 cue from the keratinocytes to execute cell conversion. ECM homeostasis, a critical cornerstone of the physiological repair process, is largely guided by the converted fate of infiltrating inflammatory cells. This work therefore introduces a novel dimension to the significance of the post-injury acute inflammatory phase.

## Methods

### Animals

LysM Cre/Gt (ROSA) 26Sor ^tm4(ACTB-tdTomato,-EGFP)Luo^/J mice express cell membrane localized red (tdTomato) fluorescence in all cells/tissue, whereas cells of keratinocyte origin express membrane-localized GFP^19^. These mice were developed by crossing Gt(ROSA)26Sor ^tm4(ACTB-tdTomato,-EGFP)Luo^/J with LysM Cre transgenic mice. Gt (ROSA) 26Sor ^tm4(ACTB-tdTomato,-EGFP)Luo^/J or ROSA^mT/mG^(Jax # 007576) is a two-color fluoresecent mouse model meant for lineage tracing. These mice express cell membrane-localized red fluorescence in cells/tissues prior to Cre recombinase, and cell membrane-localized green fluorescence in Cre recombinase expressing cells. The GFP expression is also present in future cell lineages derived from these cells. In LysM-Cre transgenic mice (Jax # 004781), myeloid specific Lysozyme M directs expression of Cre recombinase thus facilitating deletion of floxed sequences in myeloid cells as described in ref. ^19^. PCR for LysM^Cre^-Rosa^mT/mG^mice was confirmed using primers tabulated in Supplementary Table 1.

K14^cre^miR-21^fl/fl^ were obtained by cross breeding mir-21^fl/fl^ mice (kind gift from Dr. Eric Olson) with K14 Cre mice (Jax # 005107). Depletion of miR-21 was performed by intra peritonial injection of tamoxifen for 2 weeks at dose 75 mg kg^−1^ body weight. PCR for K14^cre^miR-21^fl/fl^ mice was confirmed using primers tabulated in Supplementary Table 1.

LysM^cre^miR-21^fl/fl^ were obtained by cross breeding mir-21^fl/fl^ mice with LysM Cre mice (Jax # 004781). PCR for LysM^cre^miR-21^fl/fl^ mice was confirmed using primers tabulated in Supplementary Table 1.

Eight-week-old C57BL/6 mice were purchased from Harlan laboratories.

Lepr db/db mice homozygous (BKS.Cg-m+/+Lepr db/J 482, or db/db; stock no 000642) for spontaneous mutation of the leptin receptor (Leprdb) (aged 8–10 weeks) were obtained from Jackson Laboratory, Bar Harbor, ME.

The MFG-E8 wild-type (MFG-E8^+/+^) and MFG-E8-knockout (MFG-E8^−/−^) mice were provided by Dr. S. Nagata (Osaka University Medical School).

Col1-GFP mice (pOBCol3.6GFP tpz) transgenic mice were provided by Dr. Traci Wilgus.

Act-EGFP mice display widespread EGFP fluorescence except erythrocytes and hair were obtained from Jackson laboratories (Jax#006567)

B6 Thy1.1 transgenic mice with CD90.1 allele were obtained from Jackson laboratories (Jax# 000406).

All animal studies were approved by the Ohio State University Institutional Laboratory Animal Care and Use Committee (ILACUC) under protocol 2009A0214-R2. Mice were housed under a 12-h light–dark cycle with food and water ad libitum. Mice between 8 and 10 weeks old and of both sexes were used for experiments. The animals were tagged and grouped randomly 5–6 animals in each group. No statistical method was used to predetermine the sample size. Power analysis was not necessary for this study. The randomization of animals were done using Research Randomizer software.

### Human samples

Human wound fluid were collected from chronic wound patients at OSU Comprehensive Wound Center (CWC) from the Negative Pressure Wound Therapy (NPWT) dressing (sponges) by lavaging the wound dressing with saline solution. All human studies were approved by The Ohio State University’s (OSU) Institutional Review Board (IRB) # 2007H0270—titled “Mechanism underlying impaired diabetic wound healing”—Principal Investigator, Sashwati Roy. The Declaration of Helsinki protocols were followed and patients gave their written informed consent.

### Cell lines

Human keratinocytes (HaCaT) were maintained in Dulbecco’s modified Eagle medium (DMEM; GIBCO) base medium supplemented with 10% (v/v) fetal bovine serum and 0.5% (v/v) antibiotic–antimycotic solution (GIBCO). The cells were obtained from the laboratory of Dr. NE Fusenig, Germany. The cells were checked for mycoplasma contamination using mycoplasma detection kit (R&D). In case of any contamination, mycoplasma removal agent Plasmocin (Invivogen) was used.

### Monocyte isolation from peripheral blood

Monocytes were isolated from the peripheral blood. Isolated monocytes were differentiated to macrophages using hMCSF and Polymixin B. Macrophages were polarized to M1 phenotype using LPS (1 µg/ml) and IFNγ (20 ng/ml).

### Wound model

Two 5 mm biopsy punch excisional wounds were created on the dorsal skin. The excisional wounds were made at equal distance from the midline. In order to prevent contraction of the wounds, silicon splint was used allowing wounds to heal through granulation and re-epithelialization. Mice were anesthetized by low-dose isoflurane inhalation during the wounding procedure (1–3% mixture with Oxygen) as per standard recommendation.

### Hunt/Schilling cylinder for wound fluid collection

Wire mesh cylinder (stainless steel; 2.5 cm length and 0.8 cm diameter) were implanted subcutaneouly on the dorsal side of mice. The wound fluid collected in these cylinders was harvested on d3 post implantation^19^.

### Polyvinyl alcohol sponge implantation and isolation of macrophages

Circular (8 mm) sterile polyvinyl alcohol (PVA) sponges were implanted subcutaneously on the dorsum of the mice^19^. Sub-cutaneously implanted PVA sponges were harvested on days 3 and 7 post implantation. Macrophages were isolated by magnetic cell sorting using anti-CD11b-tagged microbeads (Miltenyi Biotec, CA).

### Histology

Wound edges embedded in OCT were sectioned using a cryo microtome (Leica). The sections (10 μm) were immune stained with the following primary antibodies dilutions indicated in parenthesis: F4/80 (1:400; Biorad Cat # MCA497), FSP1 (1:400, Abcam cat# ab27957), Col1 (1:200, Abcam cat # ab19811), Col1(1:300, Abcam cat# ab34710), CD68 (1:200, Dako# M0814), PDGFRα (1:200, Abcam cat # ab61219), CD34 (1:200, Abcam cat # ab81289), MPO (1:200, Dako # A0398), Vitronectin (1:100, Abcam cat # ab45139), PTEN (1:400, Cell Signalling #9559), KLF5 (1:100, Abcam # ab137676). To enable fluorescence detection, sections were incubated with appropriate Alexa Fluor® 488 (green, Molecular probes, Eugene, OR), Alexa Fluor® 564 (red, Molecular probes) or Alexa Fluor® 405 ( uv, Molecular probes) conjugated secondary antibodies. Counter nuclear staining was done using DAPI (Sigma) or ToPro3 (Molecular probes).

### Fluorescence image analyses

Tissue sections were analyzed by fluorescence microscopy (Axiovert 200 M, Zeiss, Germany) or Confocal microscopy (Olympus). Image analysis software Zen (Zeiss) or Fluoview 2.0 (Olympus) was used to quantitate fluorescence intensity (fluorescent pixels). Additionally, a manual cell count of fluorescent positive cells in a field of view (FOV) using the cell count module in Zen (Zeiss). Cells overlapping the border of FOV were also included. For each image, five such FOVs were counted and data represented as percent positive. Masson’s trichrome and Picrosirius red staining were done using standard procedure.

### Flow cytometry analyses

The fluorescence and light-scattering properties (forward scatter and side scatter) of the cells were determined by using an Aria III flow cytometer (BD, for sorting) or LSR Fortessa (BD). Signals from cells labeled with conjugated fluorophores were detected. The following antibodies were used for different flow cytometry analysis. PE-conjugated CD90.2 (clone 30-H12, Biolegend # 105307, 2 µg/ml), FITC-conjugated F4/80 (clone BM8,eBiosciences # 11–4801–85, 0.5 µg/ml), APC-conjugated F4/80 (clone BM8, Biolegend # 123115, 0.5 µg/ml), eFluor-450 conjugated CD127 (clone A7R34, eBiosciences # 48-1271-80, 1 µg/ml), APC-conjugated CD 90.2 (clone 53-2.1, eBiosciences # 17-0902-8, 2 µg/ml), APC/Cy7-conjugated CD90.1 (clone OX-7, Biolegend # 202519, 1 µg/ml), Super Bright 600-conjugated CD19 (clone 1D3, eBiosciences# 63-0193-80, 1 µg/ml), and eFluor-450 conjugated CD3 (clone 17A2, eBiosciences # 48-0032-80, 0.5 µg/ml). Auto compensation was performed using samples stained with single flurophores. Gates were set manually. Gating strategy for the fluorophores has been shown in respective figures. BD Diva software (BD Biosciences) was used for analysis. Logarithmic scale was used to measure cell fluorescence. Appropriate IgG control fluorescence compensation was applied to avoid false positive signals.

### Bone marrow chimera

Bone marrow (BM) chimera was established as previously described^19^. For BM transplantation, BM recipient male mice (6–8 weeks old) were injected intraperitoneally with busulfan. The dose included a 1:1 solution of DMSO and deionized water (30 mg/kg/100 μl) once daily for 2 days resulting in partial ablation of the bone marrow^19^. Alternative approach of BM ablation using whole body irradiation, results in systemic toxicities. Moreover, irradiation resulted in inflammation of skin. To circumvent these issues, a widely used myelo-supressive approach for partial bone marrow ablation using busulfan was selected. Forty  eight  hours after the last dose of busulfan, donor BM cells isolated from femur were injected to recipient mice via tail vein injection (100 μl) to recipient mice. Engraftment in recipient mice was allowed to take place over a duration of 4 weeks after which engraftment was ascertained by determining the presence of GFP cells in the BM and the blood. The study utilized two types of recipient mice: (i) C57BL/6 obtained from Envigo laboratories and (ii) B6 Thy1.1 (Jackson# 000406) with CD90.1 allele.

### Laser capture microdissection

Laser microdissection and pressure catapulting was carried out using the Microlaser system from PALM Microlaser Technologies AG (Zeiss, Germany). Briefly, OCT embedded wound tissue from LysM^cre^ Rosa^mT/mG^ animals were cut into 10 μm sections using a cryo-microtome. The cut sections were then placed on polyethylene napthalate membrane glass slides (P.A.L.M. Microlaser Technologies AG, Germany)^56^. Prior to use the membrane glass slides were treated with RNAsin (Ambion) and UV-treated. The tissue sections were then cut and catapulted as described by our group^56^. GFP fluorescent cells were captured in lysis buffer provided with Cells-Direct RNA kit (Invitrogen). This was followed by RNA extraction and reverse transcription and mRNA quantification using real-time PCR were performed as described below.

### RNA isolation and qRT-PCR

Total RNA was isolated using mirVana miRNA isolation kit, according to the manufacturer’s protocol (Ambion). The total RNA contained the miRNA fraction. Specific Taqman assays were used to measure for miRNA abundance, miR-21 (Applied Biosystems) and mirVana qRT-PCR miRNA RT Kit (Applied Biosystems) were used with real-time PCR system and Taqman universal master mix. Relative quantification method was used to measure levels of miRNAs using U6 small nuclear RNA as the housekeeping. The transcription levels of other genes (mRNA) and house-keeping control GAPDH was quantified using SYBR green-I (Applied Biosystems). Primer sequences have been provided as Supplementary Table 2.

Gene expression profiling to focus on specific pathways was done using a 96-well human cancer drug resistance and metabolism PCR array, RT2 Profiler PCR array (PAHS-013ZC, Human extracellular matrix and adhesion molecules PCR Array, Qiagen, USA). The array comprised of 84 wells. Each well contained all the reagents required for the PCR reaction in addition to a primer for a single gene. MDM cells were treated with PTEN si (Dhermacon, On Target Plus L-003023) and KLF5 si (Dhermacon, On Target Plus L-013571) or control (Dhermacon, non-targeting siRNA D001810). cDNA was transcribed using a RT2 First Strand Synthesis Kit (QIAGEN). The resulting cDNA was diluted and loaded into the predesigned RT2 Profiler PCR Array (QIAGEN) plates. mRNA expression levels were quantified employing the 2^(−ΔΔct)^ relative quantification method.

### Microarray analysis

GeneChip® probe array analysis was performed on RNA extracted from d3 and d7 F4/80^+^ sorted CD11b positive wound macrophages from implanted PVA sponges as described^19^. The RNA integrity was tested using the Agilent 2100 Bioanalyzer (Agilent Technologies, Palo Alto, CA). A 100 ng of total RNA was then linearly amplified to cDNA. Followed by, 5.5 µg of cDNA was labeled and fragmented using the GeneChip® WT PLUS reagent kit (Affymetrix, CA). Labeled cDNA targets were subsequently hybridized to Affymetrix GeneChip® Mouse Transcriptome Array 1.0. The hybridization was carried out for 16 h at 45 °C rotating at 60 rpm. Post hybridization, the arrays were washed. The arrays were stained using the Fluidics Station 450 and scanned using the GeneChip Scanner 3000. Raw data was analyzed using Genespring GX 12.6 (Agilent). Additional processing of data was performed using dChip software (Harvard University). The microarray data is public and deposited in NCBI Gene Expression Omnibus (accession number GSE94400)

### Isolation of dermal fibroblast from skin

Primary dermal fibroblasts were isolated from murine skin or d10 post-wounded tissue explants. Tissues were disinfected by rinsing for 15 s in sterile 5 vol% Dettol (Reckitt Benckiser LLC, Parsippany, NJ) balanced with deionized water (18.3 MΩ). Tissues were then rinsed twice in sterile HEPES-buffered saline (Research Organics Inc., Cleveland, OH). Skin was then cut into small squares and incubated for 10 min at 37 °C in MCBD 153 medium (Sigma Aldrich, St. Louis, MO) supplemented with 3 vol% bovine pituitary extract (Gemini Bio-Products, West Sacramento, CA), 1× penicillin–streptomycin (Sigma Aldrich) and 625 U/ml collagenase type I (Worthington Biochemical Corporation, Lakewood, NJ). Tissue was then rinsed twice to remove the collagenase solution and placed into culture dishes with the dermal side down. Tissue was cultured in DME (Sigma Aldrich) supplemented with 4 vol% fetal bovine serum, insulin (5 µg/ml), epidermal growth factor (10 ng/ml), ascorbic acid-2-phosphate (0.1 mM), hydrocortisone (0.5 µg/ml) and penicillin-streptomycin (1×) for up to 7 days to allow the fibroblasts to migrate out of the dermis^30^. Subsequent IHC/flow sorting experiments were performed only on these migrated cells and not the explant.

### NanoString nCounter gene expression assay

NanoString nCounter analysis (NanoString Technologies) system performed direct detection of target molecules from a single sample using color-coded molecular barcodes, giving a digital quantification of the number of target molecules. GFP^−^CD90^+^ , GFP^+^CD90^+^ primary fibroblasts isolated from d10 wound explants. Another set of GFP^−^CD90^+^ primary fibroblast were isolated from unwounded skin. Gene expression profiling of 70 genes relating to ECM/fibroblast was performed using NanoString nCounter system. For this a total of 100 ng of mRNA from each sample  was hybridized overnight with nCounter Reporter (8 µl) probes in hybridization buffer and in excess of nCounter Capture probes (2 µl) at 65 °C for 17 h. The hybridization mixture contained target/probe complexes. This was allowed to bind to magnetic beads. The magnetic beads contained complementary sequences on the Capture Probe. Each probe will bind to its target. Excess probes were then washed out. This was followed by binding to sequences on the Reporter Probe using nCounter Prep Station. Biotinylated capture probe-bound samples were subsequently immobilized and recovered on a streptavidin-coated cartridge. Specific target molecules were then quantified using the nCounter Digital Analyzer. A CCD camera recorded the individual fluorescent barcodes and target molecules present in each sample by performing a high-density scan (325 fields of view). Images were finally processed internally into a digital format (RCC files).

### FSP1 promoter assay

FSP1 promoter assay was done as described in ref. ^28^. FSP1 promoter construct plasmid containing 1.0–1.3 kb insert, corresponding to the 5′-flanking sequence located ~1.3 kb upstream and up to 200 bp downstream of the transcription initiation site of human FSP1 (NM_002961) HPRM17407-PG04 (GeneCopoeia) inserted upstream of the GLuc reporter gene was used. M1-polarized human MDMs polarized were co-transfected with the FSP1 promoter reporter construct and miR-21 mimic using Lipofectamine LTX (Thermo Fisher Scientific). Media were collected 48 h post transfection and the secreted GLuc and SEAP activities were measured with the Secrete-Pair Dual Luminescence Assay Kit (GeneCopoeia) according to the manufacturer’s protocol using the Berthold Luminometer (Berthold Technologies). Normalized promoter activity has been presented as the ratio of GLuc to SEAP activities.

### miR-Target 3′-UTR luciferase reporter assay

miRIDIAN mimic-miR-21 or control mimic were transfected to human MDM cells differentiated to M1 macrophages, followed by transfection with KLF5-3′-UTR plasmid (HmiT017898, Genecopoeia). Luciferase assays were performed using the dual luciferase reporter assay system (Promega). Normalization was achieved by co-transfection with Renilla plasmid. Data are presented as the ratio of firefly: renilla luciferases.

### Exosome isolation from wound fluid

Wound fluid (250 µl) was mixed with 66 µl of ExoQuick exosome precipitation solution. Exosome isolation was performed according to the manufacturer’s protocol (SBI System Biosciences, USA). Briefly, the samples were incubated at 4 °C for overnight and then centrifuged at 13,000 rpm for 2 min. The protein-rich supernatant was removed, and the exosome-rich pellet was retained for RNA extraction. To block the exosome production GW4869 (Sigma) was used.

### Extracellular vesicles protein extraction and mass spectrometry

EV pellets were resuspended in 100 ml (50 ml for normal cell controls) of 50 mM ABC containing 0.5% Rapigest (Waters Corp.), sonicated 2 × 10 s, and incubated with shaking for 1 h at RT, digested with trypsin with a final concentration of 0.1% Rapigest. Sequencing grade trypsin (Promega) was added with a 1:100 (enzyme: substrate) ratio and digested O/N at 37 °C. Peptide concentration was determined by nanodrop (A280nm). Capillary-liquid chromatography-nanospray tandem mass spectrometry (Capillary-LC/MS/MS) of protein identification was carried out on a Thermo Scientific orbitrap fusion mass spectrometer. This spectrometer was equipped with an EASY-Spray™ Sources which operated in positive ion mode. In this experiment two mobile phase were used. Mobile phase A constituted of 0.1% Formic Acid in water. The mobile phase B comprised of acetonitrile (with 0.1% formic acid). Raw files were converted into a merged file (.mgf) using MS convert (ProteoWizard) in order to process the sequence information. The resulting mgf files were searched using Mascot Daemon by Matrix Science version 2.3.2 (Boston, MA). Proteomics data were summarized in scaffold and spectral counting was used for protein quantitation.

### In vivo macrophage depletion

In vivo macrophage depletion was obtained using clodronate liposomes (Clodronateliposomes.org). Mice were injected with 200 µl clodronate-loaded liposome suspension every other day for four doses. Control mice were injected with 200 μl PBS-loaded liposomes using the same schedule.

### Sircol collagen assay

Collagen levels were determined using sircol collagen assay kit (Biocolor, UK) per manufacturer’s recommendation. In brief, Sirius-Red dye in picric acid was added to cell lysate followed by vigorous shaking for 30 min. This was followed by centrifugation at 10,000×*g* for 10 min. Following centrifugation, the pellet was collected and washed with wash solution to remove the unbound dye. The pellet was dissolved in an alkaline solution and the absorbance measured using a UV-VIS plate reader at 550 nm (Synergy 2, Biotek). The collagen levels were calculated using a calibration curve that was constructed using bovine collagen-I.

### Nanoparticle-mediated delivery of miR-21

Synthesis of mannose-tagged nanoparticles (mannosylated-1,2-dioleoyl-sn-glycero-3-phosphoethanolamine(M-DOPE)) involved solubilizing α-Dmannopyranosylphenyl isothiocyanate (MITC) in DOPE/DMSO, followed by addition of *N*,*N*-diisopropylethylamine (DIPEA) and finally was purified by silica gel chromatography. The miR-21 lipid nanoparticles (LNPs) were prepared by combining DOPC/M-DOPE/Tween-80/DiO (94.9:3:2:0.1 mol/mol) followed by dissolving in ethanol. Lipids were dried (Rotavap) to remove ethanol followed by hydration and sonication to reach particle size ~800 nm. miR-21 mimic or antago-miR21 encapsulation in LNPs was then achieved followed by storing at 4 °C. Control LNP carried non-specific miR-mimic.

### Skin stiffness measurement

The mechanical properties of murine skin were quantified via uniaxial tensile testing. Murine skin was cut into uniform samples using a dog bone punch with a gauge length of 9.35 mm and a gauge width of 3 mm. Samples (*n* = 6 per group) were loaded into the grips of the mechanical tester (TestResources 100R, Shakopee, MN) with the grip to grip distance set at 11 mm and strained at a rate of 2 mm/s until failure. Load (N) vs. elongation (mm) data were plotted for each sample. Skin stiffness (N/mm) was calculated in the linear region.

### Statistical analyses

Samples were coded and data collection was performed in a blinded fashion. For in vitro experiments, data are reported as mean ± SD of four to six experiments, as indicated in respective figure legends. For animal studies, data are reported as mean ± SD of at least four to six animals, as indicated. Normal distribution of the data was checked using miniTab program (Version 17.0). Student's *t* test (two tailed) was used to determine significant differences. Comparisons among multiple groups were tested using ANOVA. A *p* value < 0.05 was considered statistically significant.

### Data availability

The microarray data have been deposited in the NCBI Gene Expression Omnibus under accession code GSE94400. All relevant data are available from the corresponding author upon reasonable request.

## Electronic supplementary material

Supplementary Information
