# Supplementary material for: Direct conversion of injury-site myeloid cells to fibroblast-like cells of granulation tissue
Source: Nat Commun. 2018 Mar 5;9:936. doi: 10.1038/s41467-018-03208-w (PMC5838200; doi:10.1038/s41467-018-03208-w)
Supplement: Supplementary file 1 — Supplementary Information [file 41467_2018_3208_MOESM1_ESM.pdf]

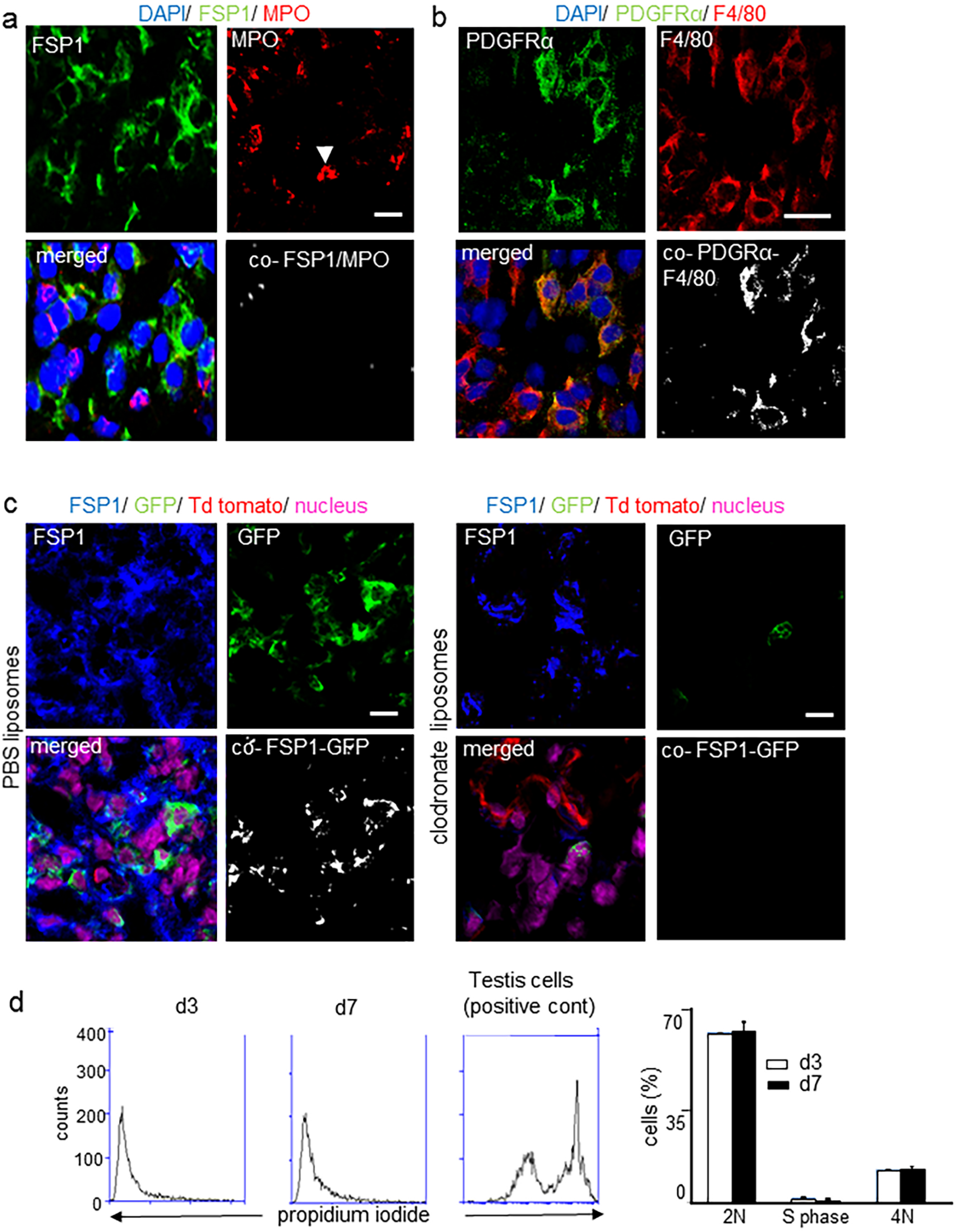

Supplementary figure 1 (**a**) α-MPO (neutrophil marker, red) and FSP1 (fibroblast marker, green) immuno-staining of d5 wound tissue from C57BL/6 mice with, n=5, scale bar =10 μm. (**b**) α-PDGRFα (fibroblast marker, green) and α-F4/80 (macrophage marker, red) immuno-staining of d5 wound tissue from C57BL/6 mice, n=5, scale bar =10 μm. (**c**) α-FSP1(blue) immuno-staining and macrophages (GFP⁺, green) co-localization of d5 wound tissue of d5 wound tissue from LysM^{Cre}Rosa$^{mT/mG}$ mice treated either with clodronate liposomes or liposomes PBS (control mice) liposomes, n=5, scale bar =10 μm. (**d**) Propidium iodide staining for DNA ploidy assay with isolated wound macrophages at d3 and d7 post- wounding, n=5.

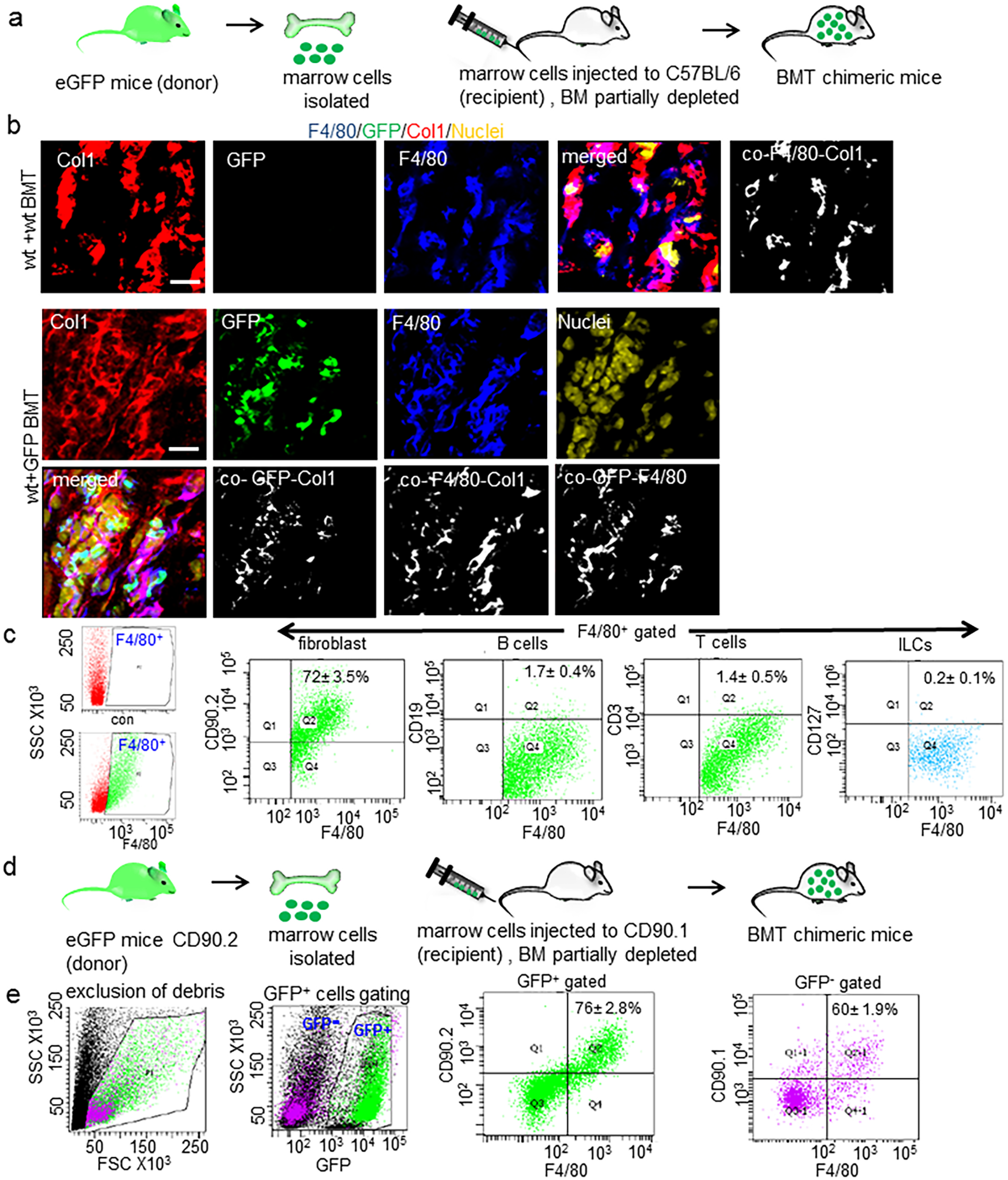

Supplementary figure 2 (a) Schematic representation of bone marrow transplantation procedure with ACTb-EGFP mice (donor) and C57bl/6 mice (recipient). **(b)** α-Col1 (fibroblast marker, red), marrow derived cells (GFP⁺, green), F4/80 (macrophages, blue) immuno-staining of d5 wound tissue from C57BL/6 mice transplanted with bone marrow of ACTb-EGFP mice, upper panel non-GFP BMT, middle and lower panels GFP BMT, n=5, scale bar =10 μm **(c)** Multicolor flow cytometry of $CD11b^{+}$ wound macrophages on d7 PW gated for $F4/80^{+}$ cells. The number of F4/80+ cells dual positive for lymphoid markers were negligible (less than<2%). % data shown in Q2 are mean±SD (n=5). **(d)** Schematic representation of bone marrow transplantation from ACTb-EGFP mice with CD90.2 allele (donor) to B6 Thy 1.1 mice with CD90.1 allele (recipient). **(e)** Multicolor flow cytometry of $CD11b^{+}$ wound macrophages on d7 PW. The GFP+ gated cells show dual positive for CD90.2 (from donor) and F4/80 (macrophages) while the GFP- gated cells display dual positive for CD90.1 (from recipient) and F4/80 (macrophages), n=4.

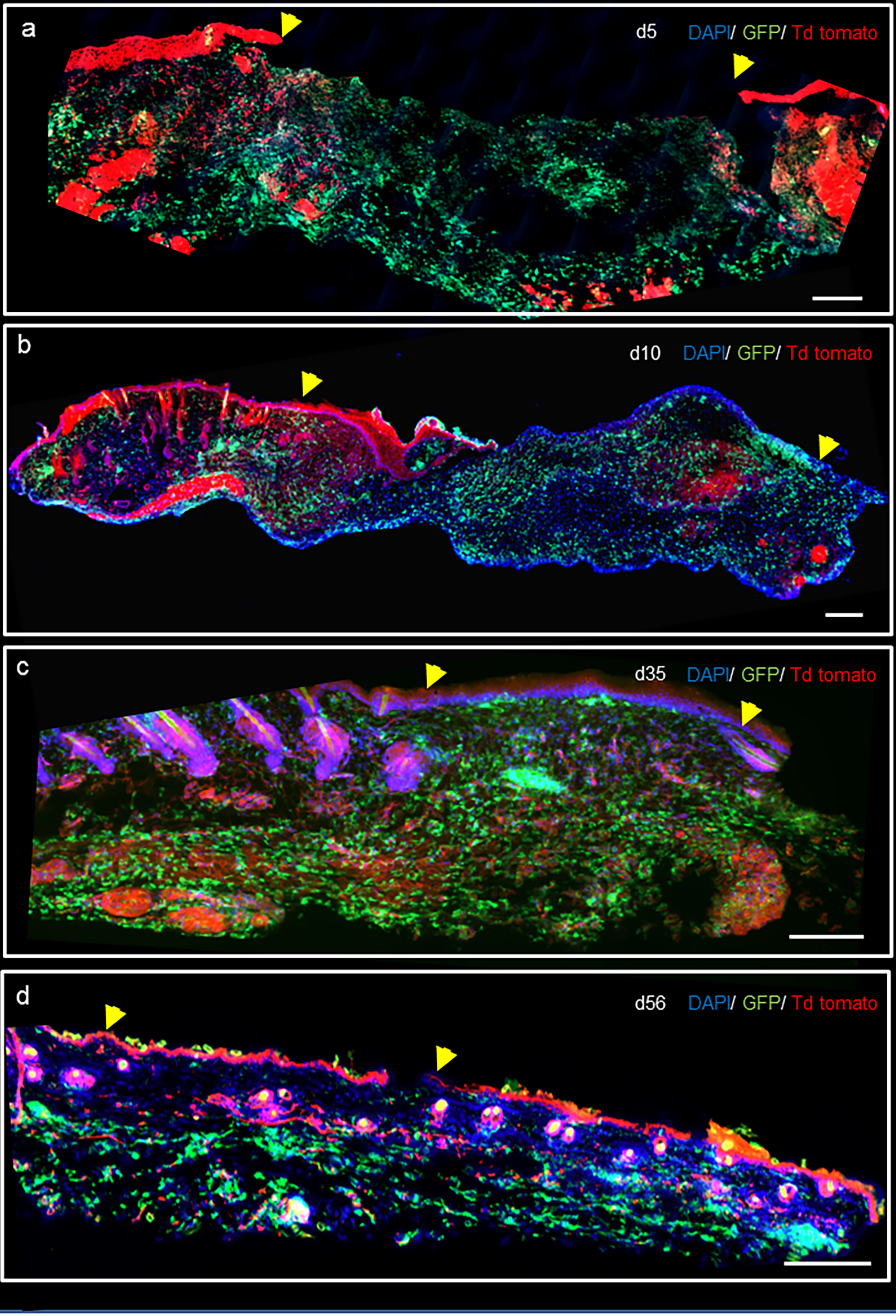

Supplementary figure3 Whole mount of wounds from LysM^{Cre}Rosa$^{mT/mG}$ mice at specified times post-wounding. Macrophage GFP^{+} (green) while the rest of the cells are TdTomato (red), wound edge shown in yellow arrow heads. scale bar = 200 µm.

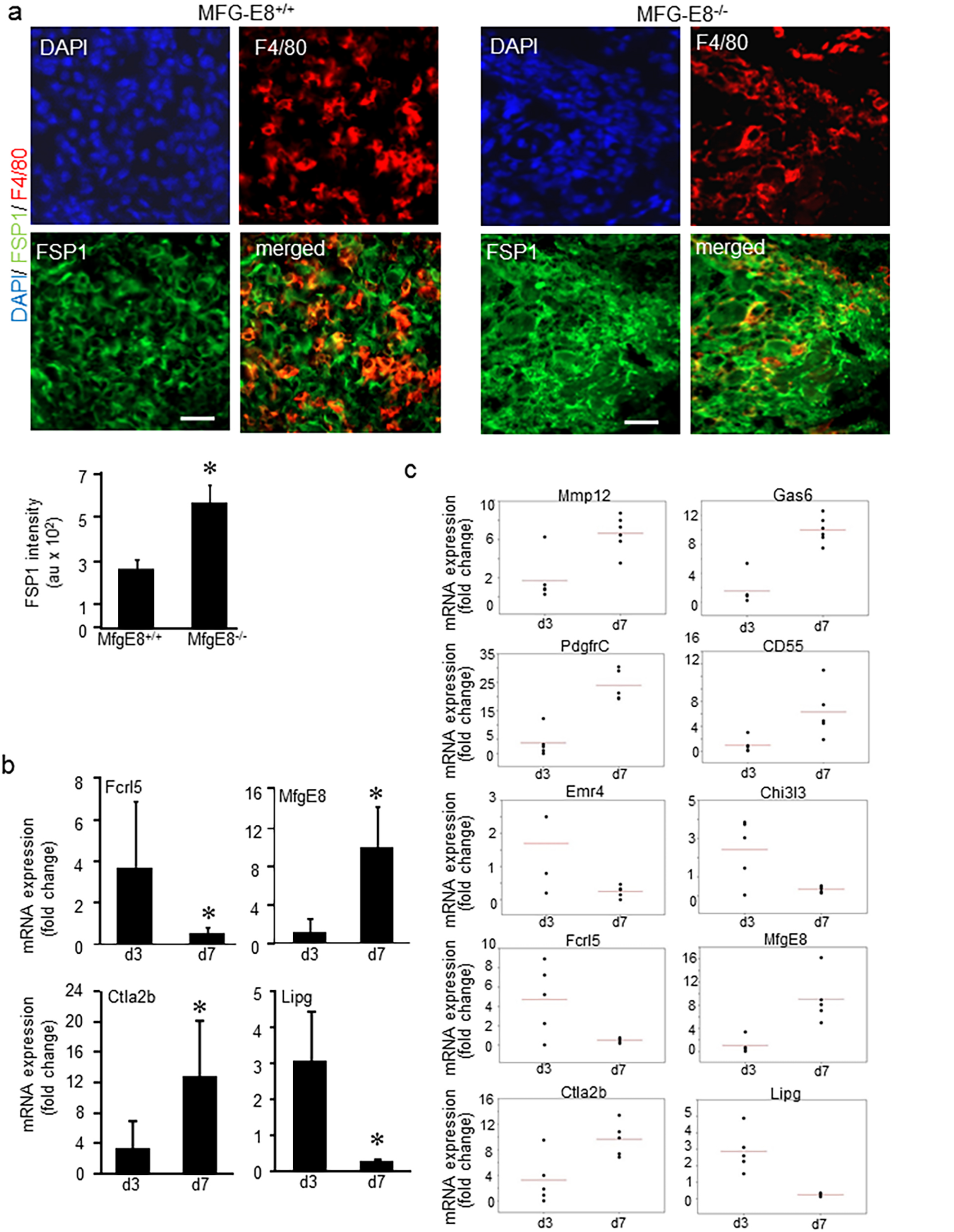

Supplementary Figure 4 (**a**) FSP1 (green) and F4/80 (red) immuno-staining of d7 wounds from MFG-E8$^{+/+}$ and MFG-E8$^{-/-}$ mice. FSP1 intensity expressed in arbitrary units, n=5, Student's t test $p<0.05$, scale bar =50 µm. (**b**) QRT PCR analysis of selected genes differentially expressed using transcriptome assay: Fclr5, MfgE8, Ctla2b and Lipg mRNA expressions, n=5, Student's t test $p<0.05$ (**c**) QRT PCR analysis where statistically significant data with overlapping SD have been replotted with individual data points (Fig 3c and Supp Fig 4b).

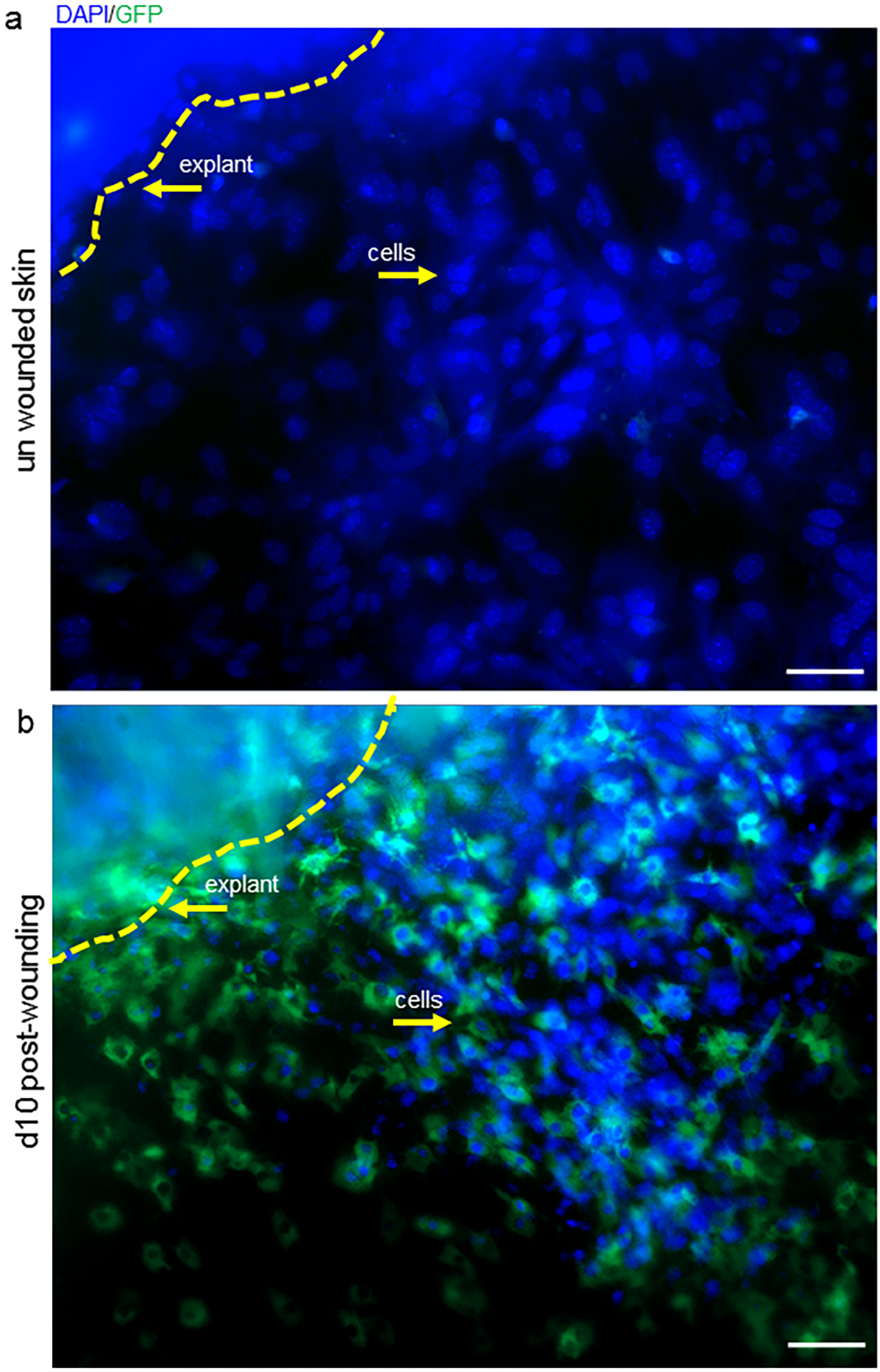

Supplementary figure 5 (a-b) Enlarged view of primary dermal fibroblasts isolated from wound/skin explants isolated of LysM^{Cre}Rosa$^{mT/mG}$ mice (**a**) un-wounded skin, (**b**) d10 post-wounding. Yellow dotted lines indicates the margin of the skin explants, n=5, scale bar =50 μm.

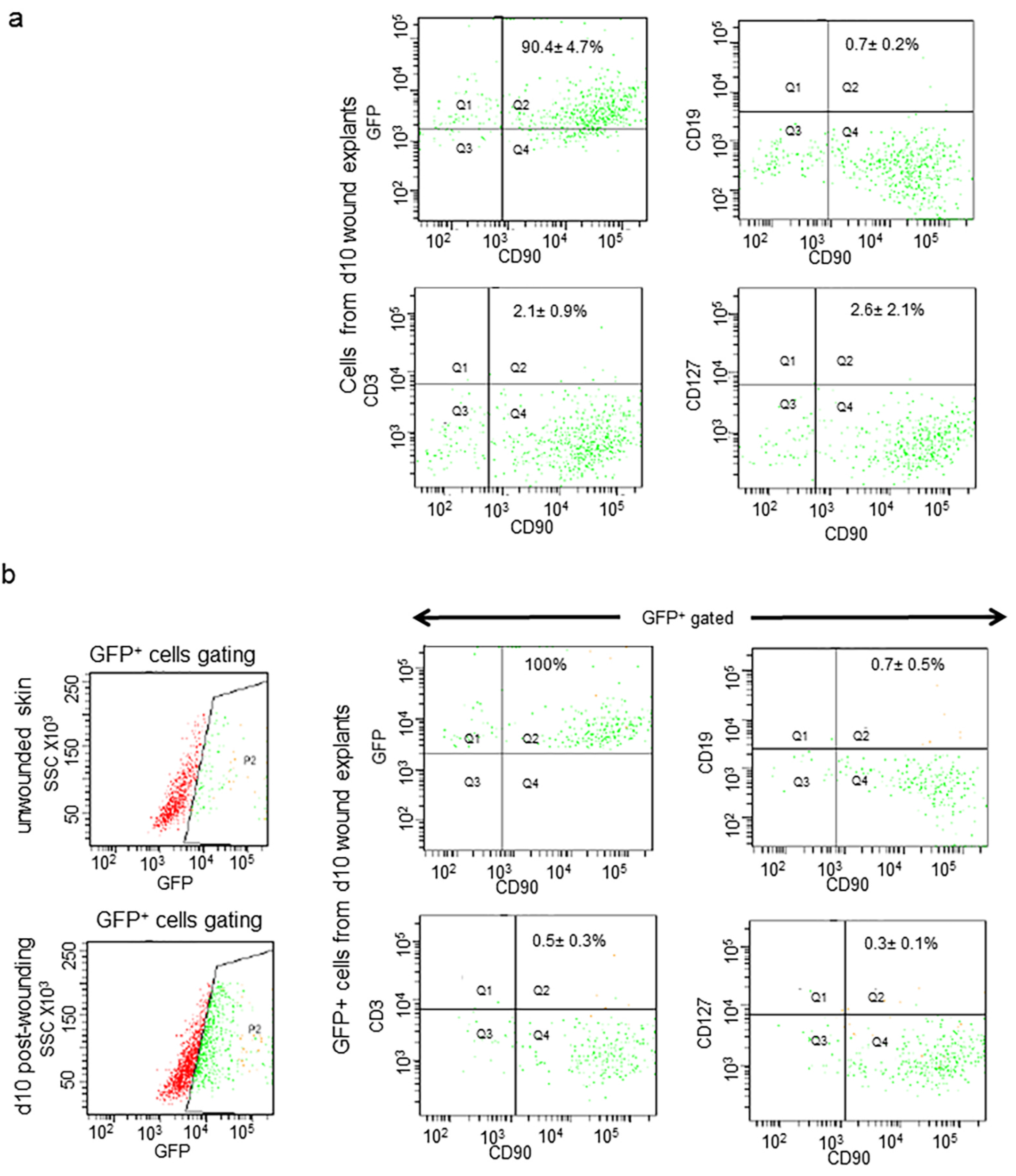

Supplementary figure 6 (a) Multicolor flow cytometry analysis of cells isolated from Rosa$^{mT/mG}$ mice d10 wound explants. The number of CD90+ cells dual positive for lymphoid markers were negligible (less than<5%). % data shown in Q2 are mean±SD (n=4). **(b)** Multicolor flow cytometry analysis of cells isolated from Rosa$^{mT/mG}$ mice d10 wound explants. The analysis was performed in GFP gated cells of myeloid origin. The number of GFP+/CD90+ cells also positive for lymphoid markers were negligible (less than<2%). % data shown in Q2 are mean±SD (n=4).

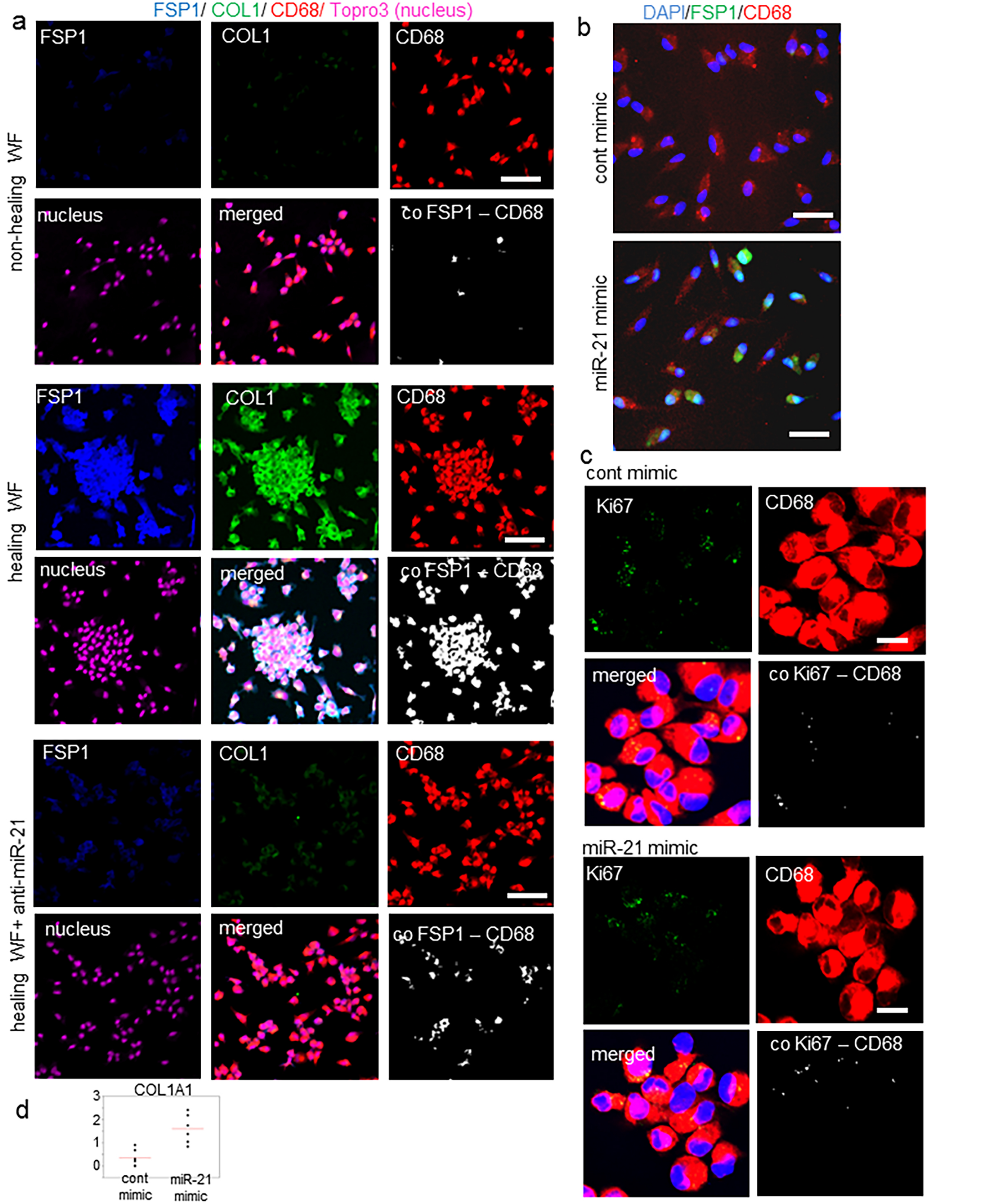

Supplementary figure 7 (**a**) MDM were polarized to M1 macrophages with LPS and IFNγ. Treatment of M1 polarized MDM ($CD68^+$, red) with fluids (WF) from non-healing wounds ($heal_{lo}$), healing wounds ($heal_{hi}$) or healing wounds + antagomiR-21 ($heal_{hi+}$antagomiR-21) for 72 h. CD68 (red), FSP1 (blue) COL1 (green) and nuclei (magenta) were determined using immuno-staining, scale bar =50 µm, n=5. (**b**) miR-21 or control mimics were delivered to by immuno-staining with CD68 (macrophage, red) and FSP1 (fibroblast, green), scale bar =100 µm (wide view of Fig 5e). (**c**) Macrophage (CD68, red) and Ki67, green immuno-staining of M1 polarized MDM followed by transfection with miR-21 mimic and scrambled oligo, n=5, p<0.05. **(d)** QRT PCR analysis where statistically significant data with overlapping SD have been replotted with individual data points (Fig 5i).

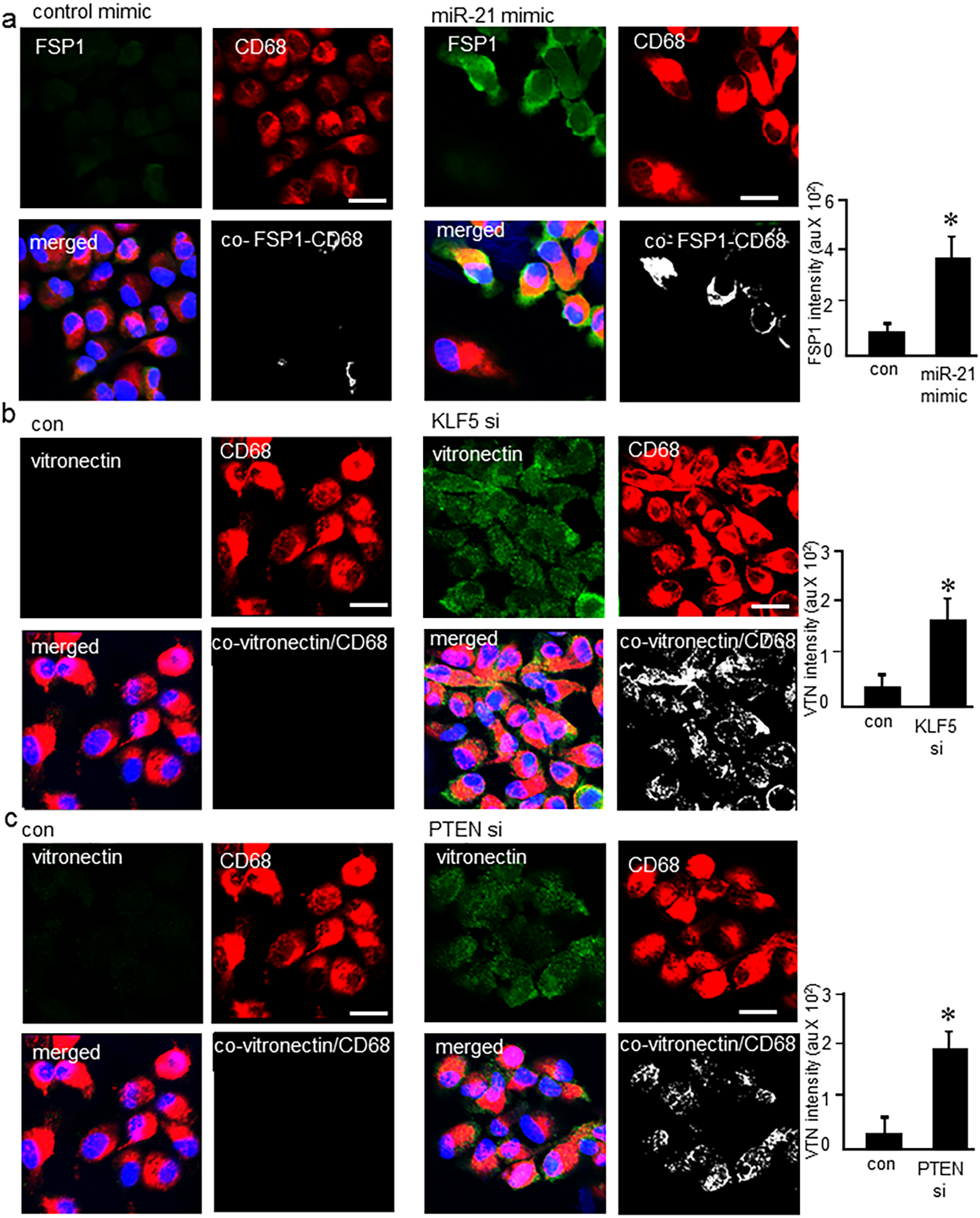

Supplementary figure 8 (a) Macrophage (CD68, red) FSP1 (green) immuno-staining of human MDM polarized to M1 phenotype followed by transfection with miR-21 mimic or scrambled oligo, n=5, $p<0.05$. Quantification of FSP1 Expression, n=6, Student's t test $p<0.05$, scale bar =10 µm. **(b)** Macrophage (CD68, red) and vitronectin (green) immuno-staining of human MDM polarized to M1 phenotype followed by transfection with PTEN si and scrambled oligo, n=6, Student's t test $p<0.05$. Vitronectin intensity analysis, n=5, Student's t test $p<0.05$, scale bar =10 µm.**(c)** Macrophage (CD68, red) and vitronectin (green) immuno-staining of M1 polarized MDM followed by transfection with miR-21 mimic and scrambled oligo, n=6, Student's t test $p<0.05$. Vitronectin intensity analysis, n=5,Student's t test $p<0.05$, scale bar =10 µm.

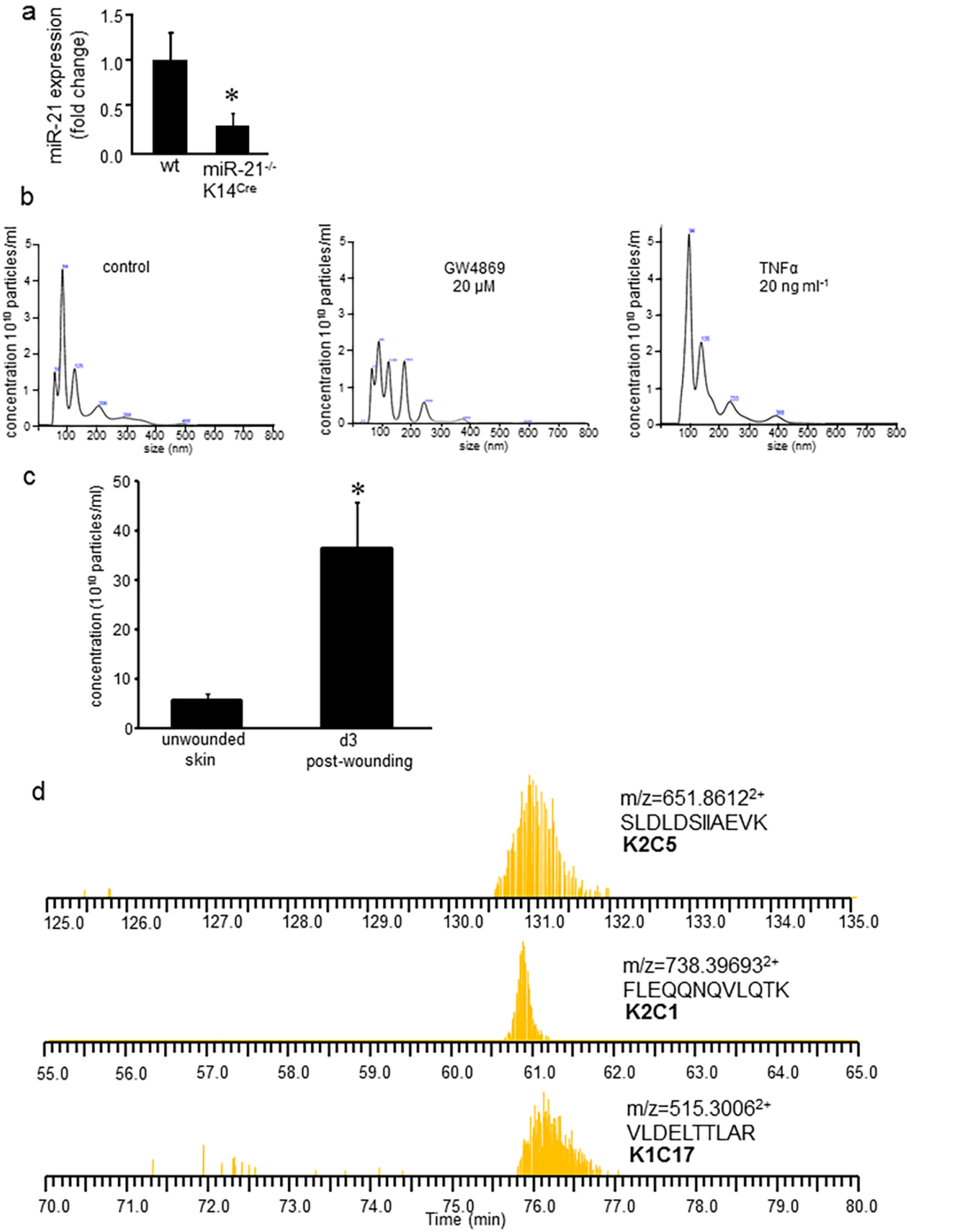

Supplementary figure 9 (**a**) Q-RT PCR analysis of miR-21 expression from laser captured keratinocytes from miR-21$^{fl/fl}$ K14^{Cre} and wt (miR-21$^{fl/fl}$) mice, n=5, $p<0.05$. **(b)** Exosome concentration from human keratinocytes (HaCaT) treated with exosome inhibitor GW4869 or inflammatory cytokine TNFα, n=6, Student's t test $p<0.05$ **(c)** Exosome concentration from unwounded murine skin or d3 post-wounding tissue, n=6, Student's t test $p<0.05$ **(d)** LC-MS peaks of keratinocyte specific proteins K2C5, K2C1 and K1C17 with corresponding peptide signatures. The proteins were identified by proteome profiling of EVs isolated from wound fluid collected in Hunt-Schilling cylinders of mice, n=5-7.

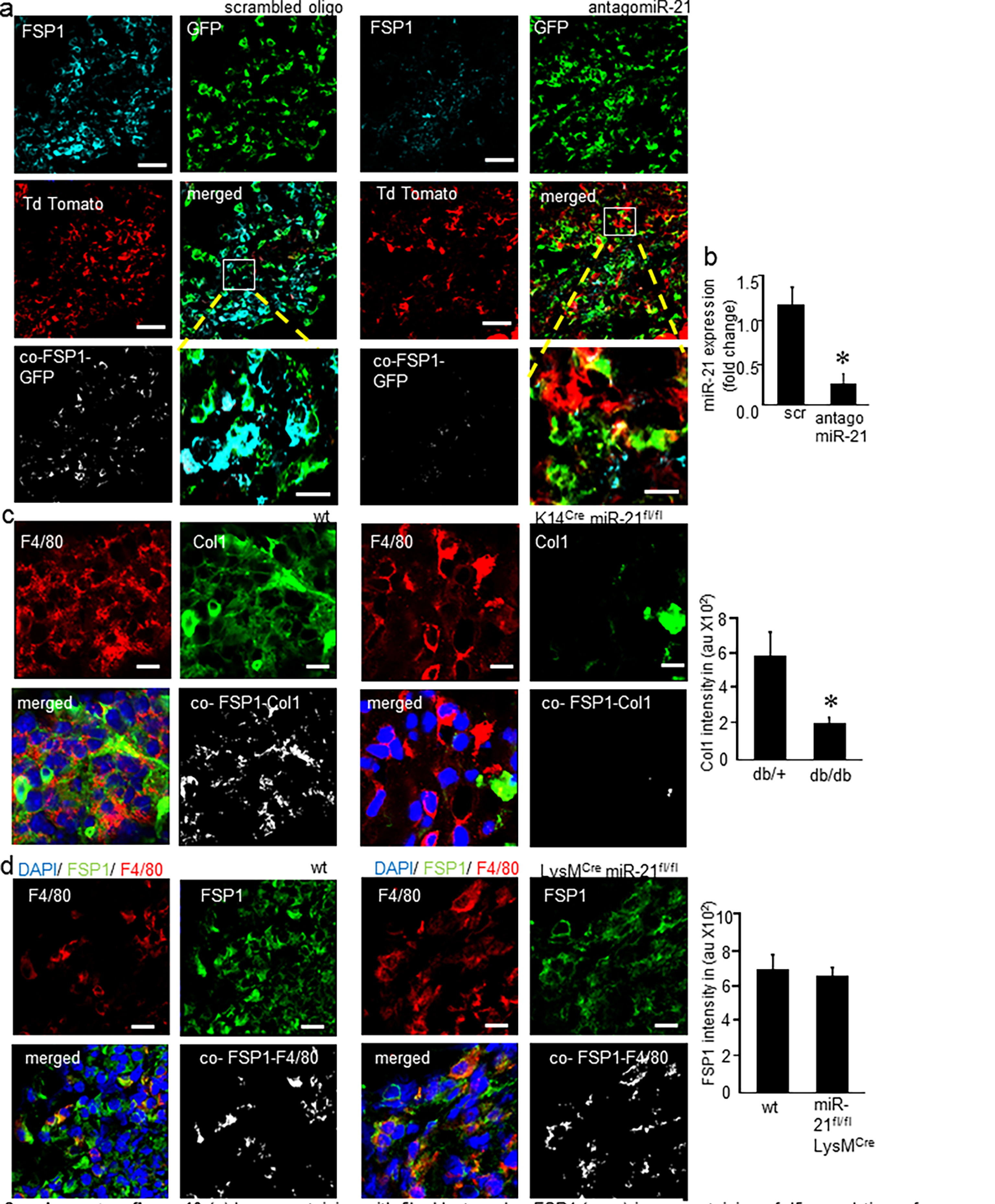

Supplementary figure 10 (**a**) Immuno-staining with fibroblast marker FSP1 (cyan) immuno-staining of d5 wound tissue from $LysM^{Cre}Rosa^{mT/mG}$ mice delivered with antagomiR-21 or scrambled oligo, scale bar =50 µm, scale bar of inset =10 µm. (b)Q-RT PCR analysis of miR-21 expression in laser captured keratinocytes from $LysM^{Cre}Rosa^{mT/mG}$ mice delivered with antagomiR-21 or scrambled oligo, n=5, $p<0.05$.(**c**) Macrophage (F4/80, red) and fibroblast (Col1, green) immuno-staining of d5 wound tissue from wt (miR-$21^{fl/fl}$) and miR-$21^{fl/fl}$ $K14^{Cre}$, n=5, $p<0.05$, scale bar =10 µm, n=5. Col1 intensity analysis in wt and miR-$21^{fl/fl}$ $K14^{Cre}$, n=5, $p<0.05$, scale bar =10 µm.(**d**) Macrophage (F4/80, red) and fibroblast (FSP1, green) immuno-staining of d5 wound tissue from wt (miR-$21^{fl/fl}$) and miR-$21^{fl/fl}$ $LysM^{Cre}$, n=5, $p<0.05$, scale bar =10 µm, n=5. FSP1 intensity analysis in wt and miR-$21^{fl/fl}$ $LysM^{Cre}$, n=5, $p<0.05$, scale bar =10 µm.

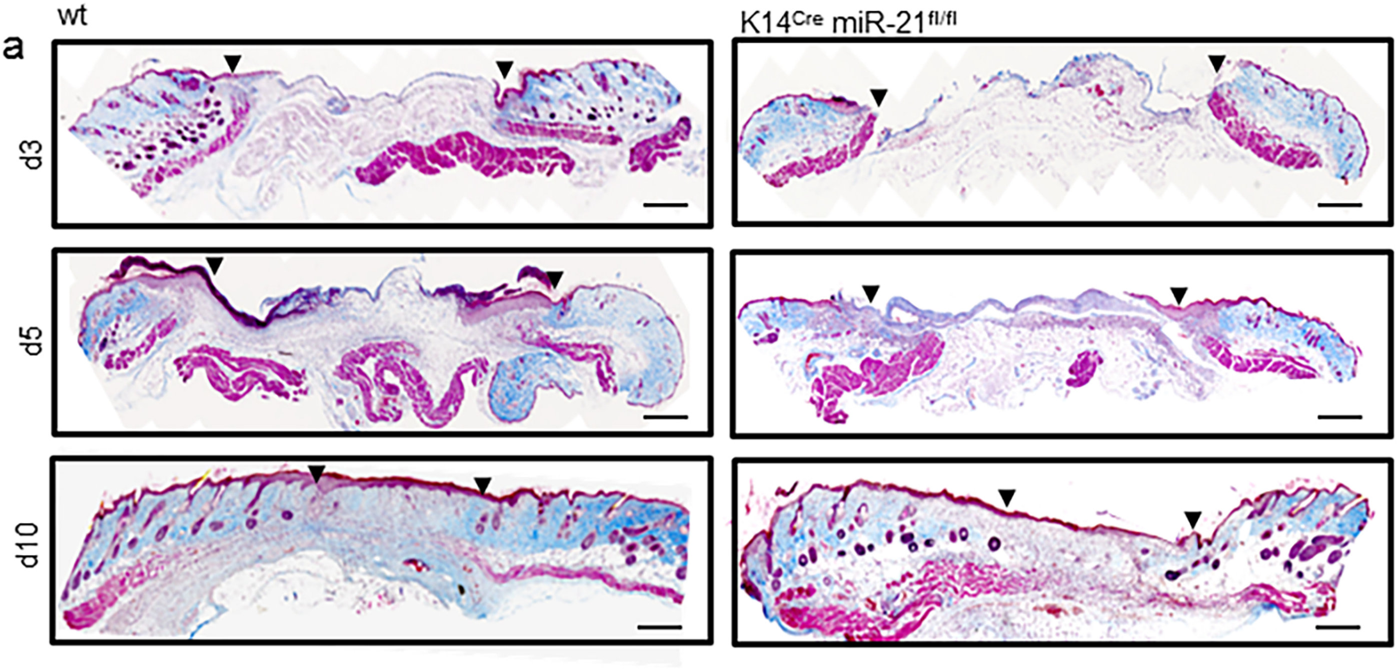

Supplementary figure 11 (a) Whole mount of Masson's Trichrome stained wounds from wt(miR-21$^{fl/fl}$) or miR-21$^{fl/fl}$ K14^{Cre} mice, n=5, wound edge shown with black arrow head, scale bar = 200 µm.

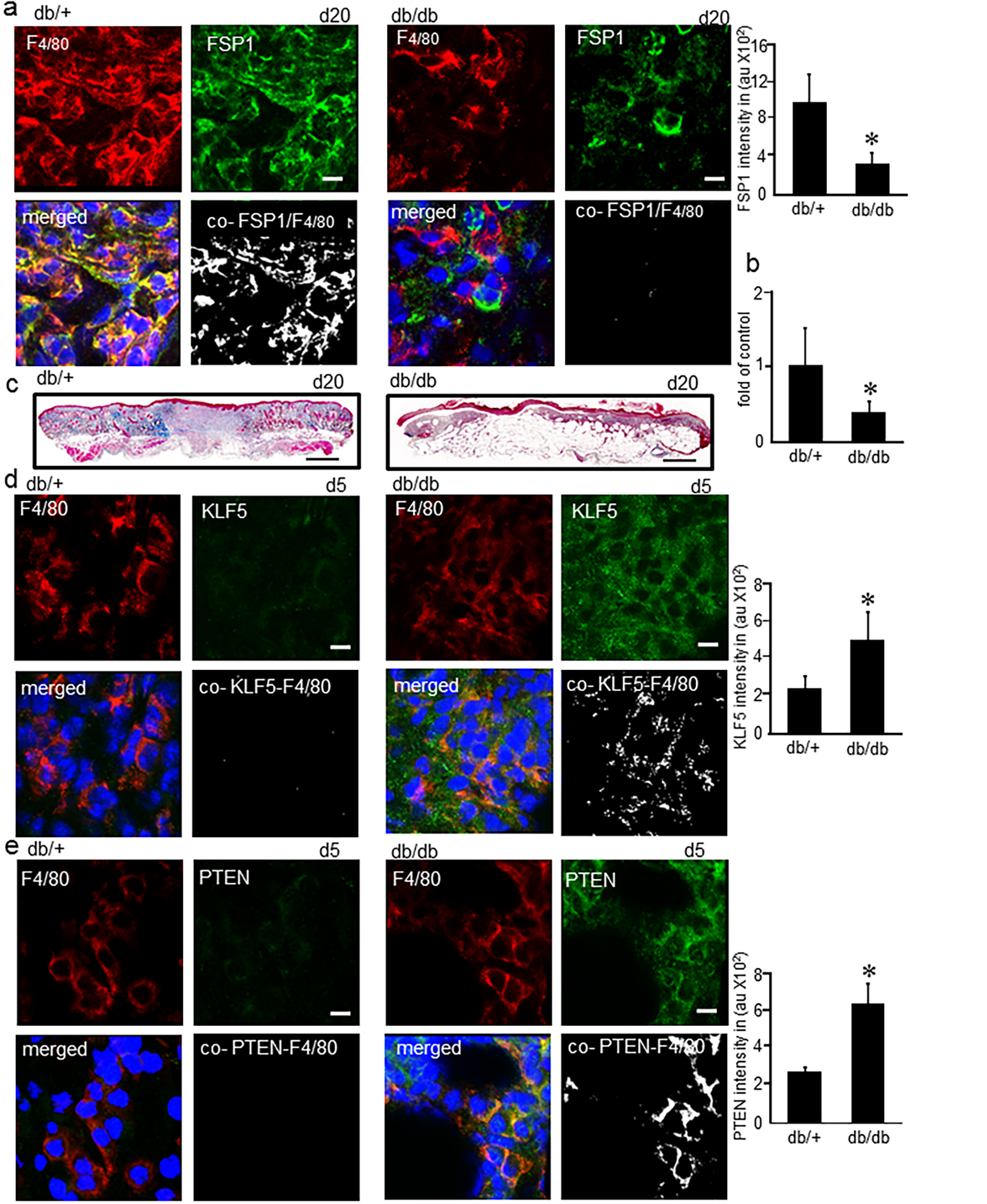

Supplementary figure 12 (**a**) Immuno-staining of F4/80 (macrophage, red) and FSP1 (fibroblast, green) of d20 wound tissue from control db/+ and db/db mice. n=5, $p<0.05$. FSP1 intensity expressed in arbitrary units. n=5, Student's t test $p<0.05$, scale bar =10 μm (**b**) Q-RT PCR analysis of miR-21 expression in wound tissue from db/db or control db/+ mice on d20 post-wounding. n=5, $p<0.05$. **(c)** Whole mounts of Masson's Trichrome stained d20 wound sections showing collagen (blue) levels in db/db and control db/+ mice, n=5, scale bar = 200 μm (**d**) Macrophage (F4/80, red) and KLF5 (green) immuno-staining in d5 wound tissue from control db/+ and db/db mice, scale bar =10 μm. Bar graphs present quantification of KLF5 in db/db and control db/+ mice, n=5, $p<0.05$(**e**) Macrophage (F4/80, red) and PTEN (green) immuno-staining of d5 wound tissue from control db/+ and db/db mice, scale bar =10 μm. Bar graphs present quantification of PTEN in db/db and control db/+ mice, n=6, $p<0.05$

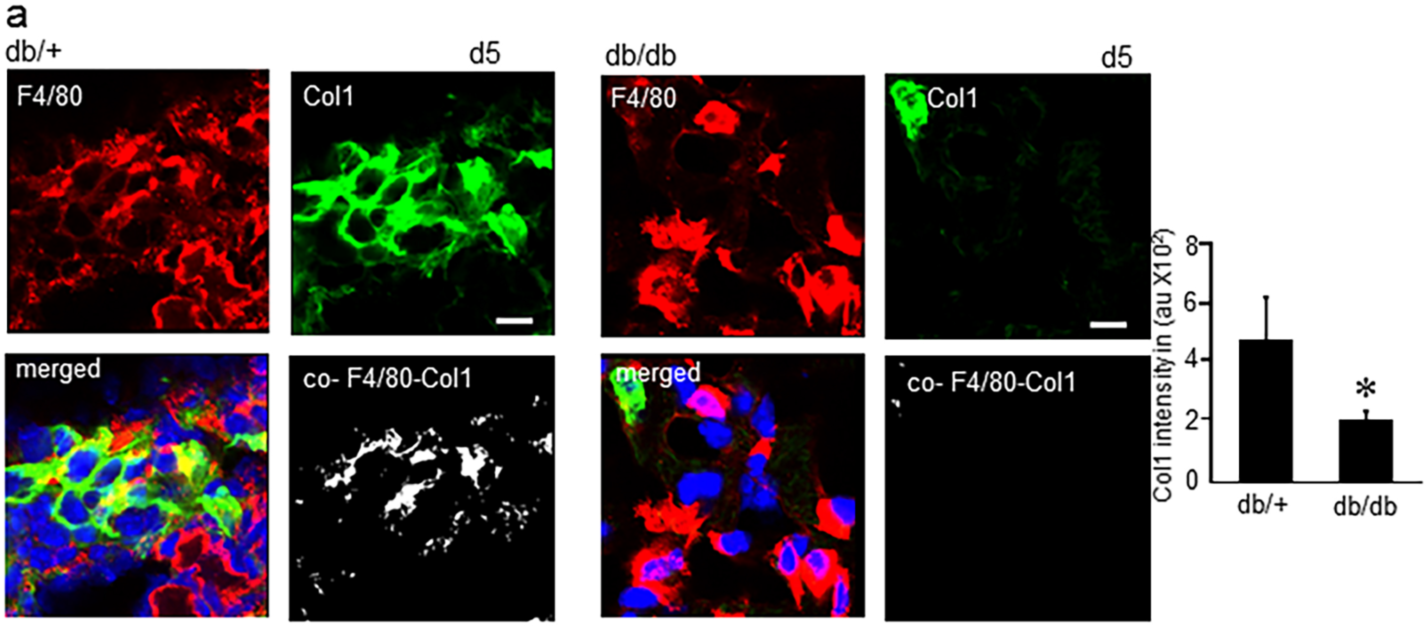

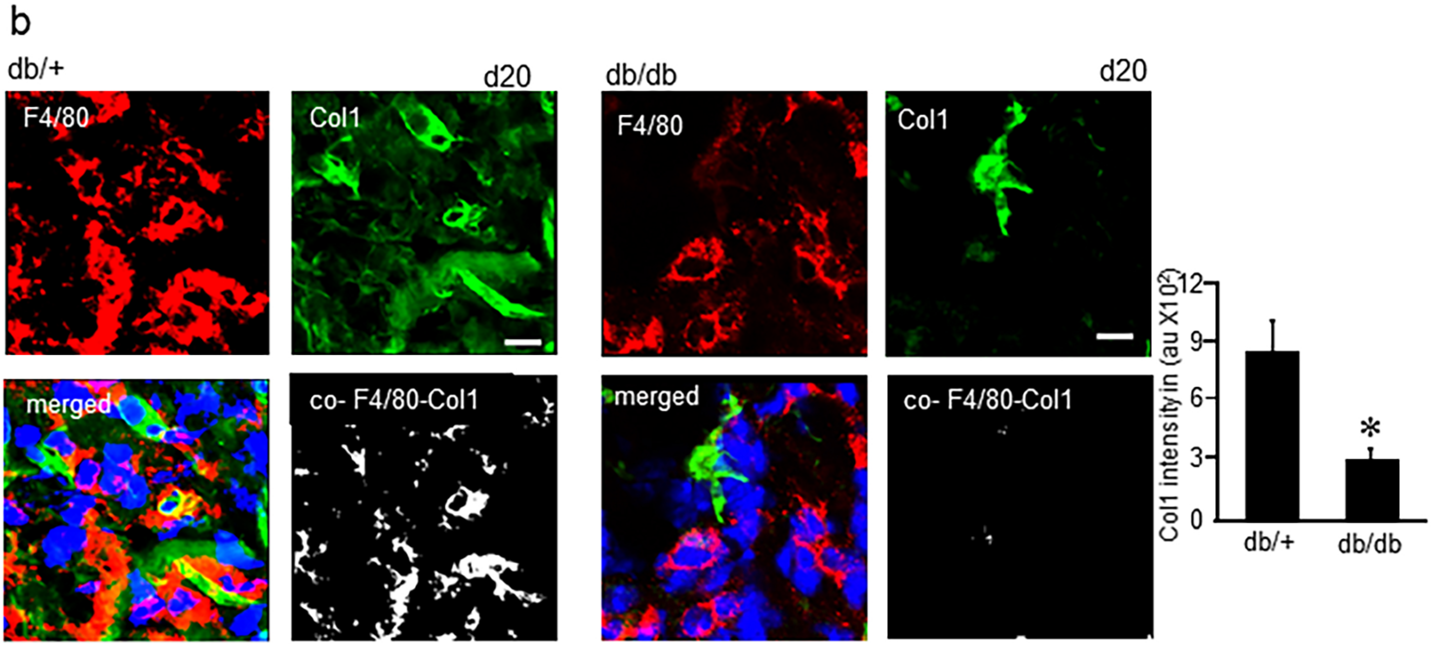

Supplementary figure 13 (**a**) Macrophage (F4/80, red) and fibroblast (Col1, green) immuno-staining in d5 wound tissue from control db/+ or db/db mice, n=5, Student's t test, $p<0.05$, scale bar =10 μm, n=5. Bar graphs present quantification of Col1a1 in control db/+ or db/db mice, n=5, $p<0.05$, scale bar =10 μm. (**b**) Macrophage (F4/80, red) and fibroblast (Col1, green) immuno-staining of d20 post-wounding from control db/+ or db/db mice, n=5, Student's t test, $p<0.05$, scale bar =10 μm, n=5. Bar graphs present quantification of Col1 analysis in control db/+ and db/db mice, n=5, Student's t test $p<0.05$, scale bar =10 μm.

Supplementary Table 1- Primer information for genotyping

		**Primer**	**Type**
	Rosa mT/mG		
	oIMR7318	CTC TGC TGC CTC CTG GCT TCT	Common forward
	oIMR7319	CGA GGC GGA TCA CAA GCA ATA	Wild type reverse
	oIMR7320	TCA ATG GGC GGG GGT CGT T	Mutant reverse
	LysM Cre		
	oIMR3066	CCC AGA AAT GCC AGA TTA CG	Mutant forward
	oIMR3067	CTT GGG CTG CCA GAA TTT CTC	Common reverse
	oIMR3068	TTA CAG TCG GCC AGG CTG AC	Wild type reverse
	K14 Cre		
	oIMR1084	GCG GTC TGG CAG TAA AAA CTA TC	Transgene forward
	oIMR1085	GTG AAA CAG CAT TGC TGT CAC TT	Transgene reverse
	miR-21		
		GCT TAC TTC TCT CTG TGA TTT CTG TG	Forward
		GGT GGT ACA GCC ATG CGA TGT CAC G	Reverse

Supplementary Table 2- Primer information for Real Time PCR, human (h), mouse (m)

	**Gene Name**	**Forward Primer**	**Reverse Primer**
	hFSP1	TCT TTC TTG GTT TGA TCC TG	GCA TCA AGC ACG TGT CTG AA
	hCol1A1	CAG GTC TCG GTC ATG GTA CCT	GTC GAG GGC CAA GAC GAA
	hGAPDH	TGA CGC TGG GGC TGG CAT TG	GCT CTT GCT GGG GCT GGT GG
	mFSP1	AGC TGC CTA GCT TCC TGG GGA	CCG GGG CTC CTT ATC TGG GCA
	mCol1a1	CCC AAT GGT GAG ACG TGG AA	TTG GGT CCC TCG ACT CCT AC
	mMMP12	GAA CTT GCA GTC GGA GGG AA	CCC AGT TGC TTC TAG CCC AA
	mGas6	ATG AAG ATC GCG GTA GCT GG	TGT TCG GGT GTA GTT GAG CC
	mPdgfrC	GCC TTG GAG TCG TCG CTT C	ATC TTG CAC TCC GTT CTG TTC C
	mCD55	CCC AGG AAA AGC AAC TTG CC	CCC TGG GTT GCA TGA GAA GT
	mEmr4	TGG AAC ACA CAA CCA CTG CT	CAA GCC AAT GCC CCA AGA AC
	mChi3l3	CCT GCC TGT GTA CTC ACC TG	CTT CCT CGA GAC CCA GGG TA

	mFcrl5	TAC CAT TGC GAG CAA GAG CA	AAA GTG GTC CAT GGA GGC TG
	mCtla2b	CAG TGC TGT CTT CCT GCT CA	CCC CGC TCA TAG TCT GCA TT
	mLipg	GGG ACA GAG AGT AGC TGG GA	CCA AAG GAC AGC GTG TAG GT
	mMfge8	ACA GGC CTC CTG CTA GCC CC	CAA GCG GAC GTG GGC CTC TC
	mGAPDH	GTG CAG TGC CAG CCT CGT CC	GCA CCG GCC TCA CCC CAT TT
